# Supplementary material for: The impact of strike action by Ghana registered nurses and midwives on the access to and utilization of healthcare services
Source: PLoS One. 2022 Oct 14;17(10):e0275661. doi: 10.1371/journal.pone.0275661 (PMC9565728; doi:10.1371/journal.pone.0275661)
Supplement: Attachment — Submitted filename: PONE-D-21-35187nk.pdf [file pone.0275661.s002.pdf]

PLOS ONE

Macro-level Impact of Strike Action by Ghana Registered Nurses and Midwives.

--Manuscript Draft--

**Manuscript Number:**	PONE-D-21-35187
**Article Type:**	Research Article
**Full Title:**	Macro-level Impact of Strike Action by Ghana Registered Nurses and Midwives.
**Short Title:**	Macro-level Impact of Strike Action by Ghana Registered Nurses and Midwives
**Corresponding Author:**	Samuel Adjorlolo<br>University of Ghana<br>Accra, GHANA
**Keywords:**	Strike, nurses and midwives, healthcare access and use, Ghana
**Abstract:**	Background : As the largest professional group, nurses and midwives play instrumental roles in healthcare delivery, supporting the smooth administration and operation of the health system. Consequently, the withdrawal of nursing and midwifery services via strike action has direct and indirect detrimental effects on access to healthcare.<br>Objective : The current study examined the impact of strike action by nurses and midwives with respect to access to and utilization of health services.<br>Method: Data were collected retrospectively using field officers (i.e., nurses and midwives) recruited from the districts or regions of Ghana. Because the strike lasted for 3 days, the data collection span three consecutive days before the strike, th[illegible]e days of the strike and three consecutive days after the strike. A total of 181 datas[illegible]om different health facilities from all the sixteen regions in Ghana was used for the descriptive analysis, focusing on mean and percentages changes before, during and after strike. Data was analysed and presented on regional basis segregated by the type of health facilities.<br>Findings : The results showed that; (1) the average number of patients or clients accessing health services reduced drastically during the strike period, compared before the strike. Majority of the regions recorded more than 70% decrease in in service use during the strike; (2) the average number of patients or clients accessing healthcare services after the strike increased by more than 100% across majority of the regions.<br>Conclusion : The study showed that strike action by nurses and midwives and negatively affected access to and utilization of healthcare services.
**Order of Authors:**	Samuel Adjorlolo
	Perpetual Ofori Ampofo
	Margaretta Gloria Chandi
	Francis Kwaku Wuni
	Ernestina Asiedu
	Vida Ami Kukula
	Sampson Opoku
	David Tenkorang-Twum
**Additional Information:**	
**Question**	**Response**
**Financial Disclosure**<br><br>Enter a financial disclosure statement that describes the sources of funding for the work included in this submission. Review the submission guidelines for detailed	Not applicable

Powered by Editorial Manager® and ProduXion Manager® from Aries Systems Corporation

requirements. View published research articles from *PLOS ONE* for specific examples.<br><br>This statement is required for submission and **will appear in the published article** if the submission is accepted. Please make sure it is accurate.<br><br>**Unfunded studies**<br>Enter: *The author(s) received no specific funding for this work.*<br><br>**Funded studies**<br>Enter a statement with the following details:<br>• Initials of the authors who received each award<br>• Grant numbers awarded to each author<br>• The full name of each funder<br>• URL of each funder website<br>• Did the sponsors or funders play any role in the study design, data collection and analysis, decision to publish, or preparation of the manuscript?<br>• **NO** - Include this sentence at the end of your statement: *The funders had no role in study design, data collection and analysis, decision to publish, or preparation of the manuscript.*<br>• **YES** - Specify the role(s) played.<br><br>* typeset	
**Competing Interests**<br><br>Use the instructions below to enter a competing interest statement for this submission. On behalf of all authors, disclose any competing interests that could be perceived to bias this work—acknowledging all financial support and any other relevant financial or non-financial competing interests.<br><br>This statement is required for submission and **will appear in the published article** if the submission is accepted. Please make sure it is accurate and that any funding sources listed in your Funding Information later in the submission form are also declared in your Financial Disclosure	None has been declared

statement.<br><br>View published research articles from [*PLOS ONE*] for specific examples.<br><br>**NO authors have competing interests**<br><br>Enter: *The authors have declared that no competing interests exist.*<br><br>**Authors with competing interests**<br><br>Enter competing interest details beginning with this statement:<br><br>*I have read the journal's policy and the authors of this manuscript have the following competing interests: [insert competing interests here]*<br><br>* typeset	
**Ethics Statement**<br><br>Enter an ethics statement for this submission. This statement is required if the study involved:<br><br>• Human participants<br>• Human specimens or tissue<br>• Vertebrate animals or cephalopods<br>• Vertebrate embryos or tissues<br>• Field research<br><br>Write "N/A" if the submission does not require an ethics statement.<br><br>General guidance is provided below. Consult the submission guidelines for detailed instructions. **Make sure that all information entered here is included in the Methods section of the manuscript.**	This is a retrospective study in which data on the number of people who utilized various healthcare services before, during and after strike action by nurses and midwives was gathered. The study did not involve direct human subject engagement. Rather, data were obtained from institutional archives as an aggregate data. The focus was on how many people visited or utilized healthcare services within the frame above without focusing on the background or demographics of users of healthcare services. The data collected were also devoid of identifying information relating to the facilities. This means that neither the facility nor clients/patients will be identified. Thus, data were fully anonymized. The data on number of people accessing healthcare services is notably a public data in Ghana. Given the nature of the study, we did not seek IRB approval. The project was underpinned by other relevant ethical considerations in research, including confidentiality, data safety and data protection. The field officers volunteered to be part of the project, despite being recommended by their regional executives. Indeed, out of the 150 persons recommended as field officers, only 112 agreed to take part in the project. The rest withdrew from the study without suffering any consequences. Access to data was restricted to the research team or other individuals supporting the project, mainly data analysts. These individuals signed a statement confirming that they would adhere to the study procedures regarding confidentiality. The data collected were analyzed as regional aggregate data to further delink the facilities from the results. The compensation for the field officers ranges from GH¢100 to GH¢200 and was proportional to the number of facilities each field officer obtained data from. The compensation was to be used for transportation, communication and other costs relating to the project.

Powered by Editorial Manager® and ProduXion Manager® from Aries Systems Corporation

**Format for specific study types**<br><br>**Human Subject Research (involving human participants and/or tissue)**<br>• Give the name of the institutional review board or ethics committee that approved the study<br>• Include the approval number and/or a statement indicating approval of this research<br>• Indicate the form of consent obtained (written/oral) or the reason that consent was not obtained (e.g. the data were analyzed anonymously)<br><br>**Animal Research (involving vertebrate animals, embryos or tissues)**<br>• Provide the name of the Institutional Animal Care and Use Committee (IACUC) or other relevant ethics board that reviewed the study protocol, and indicate whether they approved this research or granted a formal waiver of ethical approval<br>• Include an approval number if one was obtained<br>• If the study involved *non-human primates*, add *additional details* about animal welfare and steps taken to ameliorate suffering<br>• If anesthesia, euthanasia, or any kind of animal sacrifice is part of the study, include briefly which substances and/or methods were applied<br><br>**Field Research**<br><br>Include the following details if this study involves the collection of plant, animal, or other materials from a natural setting:<br>• Field permit number<br>• Name of the institution or relevant body that granted permission	
**Data Availability**<br><br>Authors are required to make all data underlying the findings described fully available, without restriction, and from the time of publication. PLOS allows rare exceptions to address legal and ethical concerns. See the PLOS Data Policy and FAQ for detailed information.	Yes - all data are fully available without restriction

A Data Availability Statement describing where the data can be found is required at submission. Your answers to this question constitute the Data Availability Statement and **will be published in the article**, if accepted.<br><br>**Important:** Stating 'data available on request from the author' is not sufficient. If your data are only available upon request, select 'No' for the first question and explain your exceptional situation in the text box.<br><br>Do the authors confirm that all data underlying the findings described in their manuscript are fully available without restriction?	
**Describe where the data may be found in full sentences. If you are copying our sample text, replace any instances of XXX with the appropriate details.**<br><br>• If the data are **held or will be held in a public repository**, include URLs, accession numbers or DOIs. If this information will only be available after acceptance, indicate this by ticking the box below. For example: *All XXX files are available from the XXX database (accession number(s) XXX, XXX.).*<br>• If the data are all contained **within the manuscript and/or Supporting Information files**, enter the following: *All relevant data are within the manuscript and its Supporting Information files.*<br>• If neither of these applies but you are able to provide **details of access elsewhere**, with or without limitations, please do so. For example:<br><br>*Data cannot be shared publicly because of [XXX]. Data are available from the XXX Institutional Data Access / Ethics Committee (contact via XXX) for researchers who meet the criteria for access to confidential data.*<br><br>*The data underlying the results presented in the study are available from (include the name of the third party*	All relevant data are within the manuscript and its Supporting Information files

and contact information or URL).<br>• This text is appropriate if the data are owned by a third party and authors do not have permission to share the data.<br><br>* typeset	
Additional data availability information:	

Powered by Editorial Manager® and ProduXion Manager® from Aries Systems Corporation

Manuscript Click here to access/download;Manuscript;Manuscript.docx

Macro-level Impact of Strike Action by Ghana Registered Nurses and Midwives

Perpetual Ofori Ampofo[1]; David Tenkorang-Twum[1,2]; Samuel Adjorlolo[3,4]; Margaretta Gloria Chandi[5]; Francis KwakuWuni[6], Ernestina Asiedu[7], Vida Ami Kukula[8], Sampson Opoku[9]

1. Ghana Registered Nurses and Midwives' Association of Ghana. Head Office, Okponglo, Accra, Ghana.

2. Department of Adult Health, School of Nursing and Midwifery, University of Ghana, Legon, Accra, Ghana.

3. Department of Mental Health, School of Nursing and Midwifery, University of Ghana, Legon, Accra, Ghana.

4. Research and Grant Institute of Ghana, P. Box LG 1004, Legon Post Office, Accra, Ghana.

5. GA West Municipal health directorate, P. O .Box AM187, Accra, Ghana.

6. Regional hospital, P.O. Box 26, Bolgatanga.

7. Department of Child and Maternal Health, School of Nursing and Midwifery, University of Ghana, Accra, Ghana.

8. Dodowa Health Research Center, Dodowa, Accra, Ghana.

9. P. O. Box KF 735. Koforidua, Eastern Region, Ghana.

Corresponding Author

Dr. Samuel Adjorlolo, Department of Mental Health, School of Nursing and Midwifery, College of Health Sciences, University of Ghana, P. O. Box LG 43, Legon, Accra, Ghana; email: sadjorlolo@ug.edu.gh

Macro-level Impact of Strike Action by Ghana Registered Nurses and Midwives

Abstract

Background: As the largest professional group, nurses and midwives play instrumental roles in healthcare delivery, supporting the smooth administration and operation of the health system. Consequently, the withdrawal of nursing and midwifery services via strike action has direct and indirect detrimental effects on access to healthcare.

Objective: The current study examined the impact of strike action by nurses and midwives with respect to access to and utilization of health services.

Method: Data were collected retrospectively using field officers (i.e., nurses and midwives) recruited from the districts or regions of Ghana. Because the strike lasted for 3 days, the data collection span three consecutive days before the strike, three days of the strike and three consecutive days after the strike. A total of 181 dataset from different health facilities from all the sixteen regions in Ghana was used for the descriptive analysis, focusing on mean and percentages changes before, during and after strike. Data was analysed and presented on regional basis segregated by the type of health facilities.

Findings: The results showed that; (1) the average number of patients or clients accessing health services reduced drastically during the strike period, compared before the strike. Majority of the regions recorded more than 70% decrease in in service use during the strike; (2) the average number of patients or clients accessing healthcare services after the strike increased by more than 100% across majority of the regions.

Conclusion: The study showed that strike action by nurses and midwives and negatively affected access to and utilization of healthcare services.

Keywords: Strike, nurses and midwives, healthcare access and use, Ghana

Introduction

The services provided by nurses and midwives are essential in the attainment of universal healthcare coverage; hence the withdrawal of such services via strike action has direct and indirect detrimental effects (Van Rensburg & van Rensburg, 2013). The current study examines the impact of strike action undertaken by members of Ghana Registered Nurses and Midwives Association (GRNMA), the umbrella body of nurses and midwives in G[illegible]a.

The International Council of Nurses (ICN) described strike as worker's cessation or break in work for the purpose of demanding a change in conditions of service from an employer after a deadlock or a means of compelling employers to listen to their request (ICN, 2011). Strike actions among healthcare workers take varying forms such as suspending general healthcare delivery services and attending only to emergencies, cessation of work for few hours or stopping work (Agyepong, Kodua, Adjei, & Adam, 2012; Manthous, 2014).

Strike action by healthcare workers is underpinned by several reasons. A cross sectional study in Nigeria reported demand for increased salaries as the main cause of strike among health workers (Oleribe et al., 2016). Similar findings have been reported in South Africa, Kenya and Ghana (Gyamfi, 2011; Van Rensburg & van Rensburg, 2013; Waithaka et al., 2020). Failure of employers to honor conditions of service agreed upon by both the employer and the health workers (Oleribe et al., 2016; Van Rensburg & van Rensburg, 2013) and manipulation of union leaders by political heads (Oleribe et al., 2016; Waithaka et al., 2020) have also been implicated in strike action by health workers. Other factors associated with strike actions include: poor working environment, demotivation among staff, delayed or unfairness in promotions and discriminations (Gyamfi, 2011; Oleribe et al., 2016; Waithaka et al., 2020). Few literature have documented solidarity with a colleague for an unlawful assault, or dismissal as reasons why health workers

may embark on strike action (Oleribe, Udofia, Oladipo, Ishola, & Taylor-Robinson, 2018; Waithaka et al., 2020). Strike actions by nurses in particular is pervasive across the African continent (Gyamfi, 2011; Oleribe et al., 2018; Waithaka et al., 2020) and is motivated by the unfairness in their conditions of service, relative to other cadres of health staff especially the medical doctors. The GRNMA, like other professional associations and labor unions, has embarked on several strike actions since 1978 as a broader strategy to obtain improved and better conditions of services for her members (Gyamfi, 2011). The longest ever strike action, lasting for six weeks (18th May to 26th June) was in 1992 and it was necessitated by the selective action by the government to approve allowances for other health professionals, namely doctors and pharmacists which excluding nurses and midwives. The most recent strike action embarked on by the GRNMA and allied health professionals recently was from September 21st to 23rd, 2020 after negotiations with the Government (employer) for improved conditions of service yielded no results.

Strike actions are generally believed to negatively impact on access to healthcare, mortality, administration of healthcare system and revenue mobilization. Restricted access to healthcare is a recipe for increased mortality and morbidity and decreased revenue mobilization. This suggest that it is extremely important to examine access to healthcare as the foremost impact of strike action by healthcare workers. A study conducted in Kenya found that outpatient attendance declined by 64.4%, special clinics attendance by 74.2%, deliveries by 53.5% and inpatient admissions by 57.8% when nurses and other healthcare professionals embarked on strike (Njuguna, 2015). Another study by the same author sought to determine the effect of nurses' strike on immunization services in Kenya. Data on number of fully immunized infants reported in 18 county referral hospitals was extracted from the Kenya Health Information System and analyzed

for strike and similar non-strike months. A similar analysis was conducted for 14 faith-based health facilities that were not affected by the strike. In another study from Kenya, Njuguna (2018) found that immunization services declined by 56.9% during the strike action by nurses while Faith-based health facilities that were not affected by the strike action recorded 251.6% increase in immunizations during the strike period. A study from Kenya has also shown that pregnant women were 17% less likely to attend at least four ANC visits during their pregnancy, 16% less likely to deliver in a health facility during strike by nurses (Scanlon et al., 2021). The administration of first polio vaccine to children was negatively affected by strike nurses. Children born in the midst of strike by nurses took a longer time to receive their first dose of polio vaccine, compared with those who were not born during strike period (13 days versus 7 days)(Scanlon et al., 2021)(Scanlon et al., 2021). In Nigeria, Aturaka et al. (2018) reported that only 8% of healthcare services were accessible to the public when healthcare workers embarked on strike.

Similar findings have been reported in high income countries, including US (Sheahan, Kennedy, Tompkins, Repp, & Gilbert, 2021), England (Furnivall, Bottle, & Aylin, 2018; Ruiz, Bottle, & Aylin, 2013) and Finland (Aro & Hosia, 1987). For example, Ruiz et al. (2013) in their study in England found that compared with the non-strike period, emergency admissions fell by 2.4% while the elective admissions decreased by 12.8%. The authors reported a 7.8% drop in the number of outpatients seen by medical staff on the day of the strike and a 45.5% increase in the number of cancelled appointments by NHS hospitals, while accident and emergency attendances dropped by 4.7% (Ruiz et al., 2013).

As noted above, strike actions generally leads to a reduction in the number of people accessing healthcare services (Bhuiyan & Machowski, 2012; Oleribe et al., 2018). However, our understanding of the extent of disruption in access to a range of healthcare services is limited as

majority of previous studies have tended to focus their attention on services rendered by a unit or department in hospital or health facility (exceptions see Njuguna, 2015). Understanding how strike actions impact the range of healthcare services available to the general public will equip stakeholders to take appropriate steps to respond appropriately and timely to strike notices, avert strike actions in an attempt to safeguard the health of the population. Second, although nurses and midwives and other health professionals in Ghana do embark on strike, there is no comprehensive and systematically generated evidence on the impact of their strike actions on access to healthcare. The lack of empirical data in this regard makes it extremely difficult for the nurses and midwives association (i.e., GRNMA) to quantify the contributions of her members to healthcare delivery and to make a compelling case for better conditions of service. At the same time, the employer is unable to readily envisage the devastation to access to healthcare delivery occasioned by the strike action of nurses and midwives and other health professionals. The current study fills this void by investigating the impact of the 2020 strike action by GRNMA and its Allied health professionals on access to a range of healthcare services in Ghana.

Study Objectives

The objectives of the study are as follows;

1. Determine the number of people accessing and utilization health services before, during and after strike action by nurses and midwives.
2. Compare the number of people who accessed essential healthcare services before, during and after the strike was called off.

Method

Project Setting

Data were gathered from health facilities located across the 16 regions in Ghana and across all the levels of healthcare delivery. The healthcare system in Ghana is structured. At the highest level is the teaching hospitals, followed by regional hospitals, district hospital, polyclinic, health centers or clinic and community health planning and services (CHPS). Data were collected from health facilities that are under the auspices of Ministry of Health and Ghana Health Service (i.e., Government facility or CHAG facility). Private health facilities were excluded from the study since the working conditions of nurses at these facilities are not arranged or fall under the purview of MoH and or GHS. In this study, majority of the facilities were owned by government (n = 155, 85.6%)

Recruiting and Training Field Officers

A total of 140 field officers were recruited across the 16 regions in Ghana through the regional offices of the GRNMA. The regional officers of the GRNMA were tasked to identify at a least a field officer from each District under their jurisdiction. A total of 150 names were submitted to the research team as potential field officers. A dedicated WhatsApp platform was created for the field officers and the research team to facilitate communication relating to the project. A training workshop was organized and held via the Zoom videoconference platform at the convenience of the field officers who were working as nurses in their respective health facilities. A separate training session was organized for those who could not join the first training session. The field officers were taking through the study purpose, objectives and goals and more importantly the data gathering process. The training was designed to ensure quality data gathering and transmission via a dedicated electronic portal powered by Google. Consequently, the training focused on how to maintain data integrity, avoid data contamination as well as ensure ethically

responsible research conduct. The training session offered the participant[illegible]e opportunity to ask questions pertaining to the project. The field officers were engaged throughout the period of data collection via the WhatsApp platform.

Study Variables

The study variables, operationalized as the services rendered by the health facilities, were decided by the research team members following a series of meetings and consultations with researchers, policy makers and practitioner. The team also took into consideration local health priorities and the demands of the sustainable development goal 3. The research team unanimously agreed on the following study variables; (1) outpatient department services, (2) admissions, (3) deliveries, (4) surgical services, (5) reproductive health services, and (6) antenatal clinic services. The inclusion of delivery services, for instance, was in accordance with indicator 3.1 of the SGD3 (reducing "global maternal mortality ratio to less than 70 per 100,000 live births") and indicator 3.2 (reducing neonatal mortality to at least as low 12 per 1,000 live births……"). Reproductive health service was also included in view of indicator 3.7 of the SDG3: "universal access to sexual and reproductive health care services, including family planning……". Lastly, the focus on antenatal services reflect indicator 3.1 of the SGD3 which is to reduce "global maternal mortality ratio to less than 70 per 100,000 live births" and indicator 3.2 that is concerned about reducing neonatal mortality to at least as low 12 per 1,000 live births and under-5 mortality to at least as low as 25 per 1,000 live births."

Timeframe for Project Data

Data were collected before, during and after the strike period to allow for comparative analyses and discussions across different data collection points, with reference to the strike period. Because the strike lasted for three consecutive days (21st to 23rd September, 2020; Monday to

Wednesday), it was agreed that data should be collected for the 3-day period so that we are able to appreciate the scale of the impact of the strike. Choosing one of the three days for data collection might be challenging given that the number of clients or patients accessing healthcare services differ from day to day. To obtain a baseline data against which to assess the impact of the strike, a decision was reached to collect data for the 3 consecutive days before the strike (14th to 16th September 2020; Monday to Wednesday). Likewise, to estimate the use of health services following the suspension of the strike, data were collected 3 consecutive days after the strike was called off (28th to 30th September 2020; Monday to Wednesday). The data collection lasted for approximately 2 months, spanning 10th October to 3rd December 2020.

Data collection Procedure

Data were collected by the field officers using a data collection tool designed by the research team based on the pre-determined study variables discussed previously. Prior to the data collection, institutional permission was sought from the various health facilities with introductory or permission letter issued by the GRNMA national secretariat to the field officers. As stated previously, data were collected for three consecutive days for each data collection period (e.g., before, during and after strike). The field officers completed the hardcopy of the questionnaire for each facility. Thereafter, they were provided with a dedicated link, powered by Google Form, where they inputted and transmitted the data electronically to a centralized receiver accessible to the research team. The same questionnaire was used across the health facilities. The field officers were informed to input nil or zero where the data sought for does not exist. For example, because CHPS compounds do not conduct surgeries, data on this service will not be available. The electronic form requires that the field officers provided additional information on the region, district, type of facility (e.g., hospital or health center) and ownership of facilities (e.g.,

Government or CHAG) where data were collected. Regular updates were provided on the WhatsApp page to keep the field officers informed about the submissions received.

Ethical Consideration

This is a retrospective study in which data on the number of people who utilized various healthcare services before, during and after strike action by nurses and midwives was gathered. The study did not involve direct human subject engagement. Rather, data were obtained from institutional archives as an aggregate data. The focus was on how many people visited or utilized healthcare services within the frame above without focusing on the background or demographics of users of healthcare services. The data collected were also devoid of identifying information relating to the facilities. This means that neither the facility nor clients/patients will be identified. Thus, data were fully anonymized. The data on number of people accessing healthcare services is notably a public data in Ghana. Given the nature of the study, we did not seek IRB approval. The project was underpinned by other relevant ethical considerations in research, including confidentiality, data safety and data protection. The field officers volunteered to be part of the project, despite being recommended by their regional executives. Indeed, out of the 150 persons recommended as field officers, only 112 agreed to take part in the project. The rest withdrew from the study without suffering any consequence[illegible]ccess to data was restricted to the research team or other individuals supporting the project, mainly data analysts. These individuals signed a statement confirming that they would adhere to the study procedures regarding confidentiality. The data collected were analyzed as regional aggregate data to further delink the facilities from the results. The compensation for the field officers ranges from GH¢100 to GH¢200 and was proportional to the number of facilities each field officer obtained data from. The compensation was to be used for transportation, communication and other costs relating to the project.

Data Management

By the end of the data collection process, a total of 191 submissions were received. However, some of the submissions were duplicates, perhaps because of the technical and internet connectivity issues. It was also observed that, some field officers did not provide the exact or absolute number of service users for the study variable. Instead, they provided inscription such as “over 400”, making it difficult to determine the exact number of service users under reference. The dataset was subsequently cleaned by deleting the data anomalies or deviations, leaving a total of 181 submissions from health facilities across the Districts and regions for analyses.

Data Analysis

The analysis of data proceeded on two key assumptions; (1) the health facilities under the various categorization (e.g., hospital, health centers) in a region will be similar with respect to the average number of service users than those outside the region. That is, hospitals in Ashanti region will be similar in the number of service users, comprising patients and clients, than hospitals in Volta region. This assumption is centered heavily on the variations in the population distribution across the regions which in turn influence the number of health service consumers; and (2) the health facilities falling under a particular category will be similar in terms of the range of the services provided. For example, it was assumed that CHPS compound across the country will offer virtually the same type of health services. In the same vein, hospitals across the country are more likely to render the same set of health services. Any difference should be subtle or negligible. Based on the foregoing, data was analyzed at the health facilities level, segregated by region. That is, hospital data were analyzed on regional basis as were data from health centers.

To proceed, we computed the average number or mean of service consumers for the health facility type for each region. For example, the data from the hospitals in Ashanti region were

summed and divided by the total number of hospitals that provided the data to obtain the average number of health services from hospitals in Ashanti region. In instances where data were available for only one type of health facility in a region, the same data were used since the mean could not be calculated. Although the mean is sensitive to outliers, it is the most widely used descriptive statistics in research and publication. To address problems relating to outliers, we aggregated and analyzed data along regional framework and by nature of health facilities. Data was prepared using the IBM SPSS Version 23 and analyzed using excel. The analyses involves mostly descriptive statistics. We computed the percentage change in the average number of individuals accessing health services before, during and after strike.

Results

Distribution of Health Facilities

As noted previously, data were collected from a total of 181 health facilities. More than half of the facilities were hospitals (n = 93; 51.4%), 64 were health centers (35.4%), 16 were CHPS compounds (8.8%) and 8 were polyclinics (4.4%). Table 1 showed the regional breakdown of the number of health facilities included in the project. With respect to the 93 hospitals, majority were in Ashanti region (*n* = 12), followed by Volta and Upper West (*n* = 9 each) and Upper East and Western (*n* = 8 each), with only in Ahafo region. Of the 64 health centers, majority were in Ashanti region (n = 12), followed by Central region (n = 13) and Upper West (n = 8), with only from Northern region. Of the 16 CHPS compounds, Upper West region contributed to six, followed by Western region (n = 4). There was no data from CHPS compounds located in several regions, including Ashanti, Oti, Bono, Eastern and Ahafo. Data were obtained from two polyclinics in Greater Accra and one each in the following regions; Ashanti, Western North, Bono, Upper West, North East and Central.

Table 1: *Number and type of health facilities across the regions*

Regions	Frequency	Percentage (%)	Regions	Frequenc y	Percentage (%)
**Ashanti**			**Greater Accra**		
Hospital	12	48	Hospital	6	54.5
Polyclinic	1	4	Polyclinic	2	18.2
Health Center	12	48	Health Center	2	18.2
CHPS Compound	0	0	CHPS Compound	1	9.1
**Total**	**25**	**100**	**Total**	**11**	**100**
**Eastern**			**Ahafo**		
Hospital	5	100	Hospital	1	100
**Total**	**5**	**100**	**Total**	**1**	**100**
**Western**			**Western North**		
Hospital	8	53.3	Hospital	4	36.4
Polyclinic	0	0	Polyclinic	1	9.1
Health Center	3	20	Health Center	5	45.5
CHPS Compound	4	26.7	CHPS Compound	1	9.1
**Total**	**15**	**100**	**Total**	**11**	**100**
**Oti**			**Bono East**		
Hospital	4	100	Hospital	3	100
**Total**	**4**	**100**	**Total**	**3**	**100**
**Volta**			**Bono**		
Hospital	9	81.8	Hospital	6	66.7
Polyclinic	0	0	Polyclinic	1	11.1
Health Center	2	18.2	Health Center	2	22.2
**Total**	**11**	**100**	**Total**	**9**	**100**
**Northern**			**Savannah**		
Hospital	7	87.5	Hospital	2	18.2
Health Center	1	12.5	Health Center	6	54.5
CHPS Compound	0		CHPS Compound	3	27.3
**Total**	**8**	**100**	**Total**	**11**	**100**

Upper West			Upper East		
Hospital	9	37.5	Hospital	8	66.7
Polyclinic	1	4.2	Polyclinic	0	0
Health Center	8	33.3	Health Center	4	33.3
CHPS Compound	6	25.0	CHPS Compound	0	0
**Total**	**24**	**100**	**Total**	**12**	**100**
**Central**			**North East**		
Hospital	7	31.8	Hospital	2	22.2
Polyclinic	1	4.5	Polyclinic	1	11.1
Health Center	13	59.1	Health Center	6	66.7
CHPS Compound	1	4.5	CHPS Compound	0	0
**Total**	**22**	**100**	**Total**	9	100

Access to Health Services at Hospitals Before, During and After Strike

The impact of the strike action on access to health services at the hospitals across the 16 regions were summarized below;

Outpatient Department Attendance

As shown in Table 2, the average number of patients accessing healthcare services at the OPD reduced significantly during the strike, compared with before the strike. Taking Ahafo region as an example, it can be observed that prior to the strike, an average of 363 patients attended the OPD. This number reduced drastically to 41 during the strike period. In general, the percentage decrease in the mean OPD attendance for the regions ranges from 45.12% (Oti region) to 97.95% (North East region), whereas the percentage increase in mean OPD attendance after the strike was called off ranges from 83.40% (Bono region) to +100% (e.g., Ashanti region)

Table 2: *Percentage change in the mean OPD attendance during and after strike*

**Region**	**Before Strike**	**During Strike**		**After Strike**	
	Mean OPD	Mean OPD Attendance	% Δ from Before Strike	Mean OPD Attendance	% Δ from During Strike
Ahafo	363	41	-88.70	441	+100
Ashanti	341.67	163.92	-52.02	339.67	+100
Bono	551.83	268.17	-51.40	491.83	83.40
Bono East	702	383	-45.44	712.67	86.08
Central	370.14	116.86	-68.43	404.14	+100
Eastern	426.8	159.6	-62.61	477	+100
Greater Accra	425.33	81	-80.96	464.17	+100

North East	317.5	6.5	-97.95	341.5	+100
Northern	475	26.57	-94.40	660.14	+100
Oti	333.5	183	-45.12	361.5	97.54
Savannah	261	87	-66.67	314.5	+100
Upper East	293.38	100.63	-65.70	311.13	+100
Upper West	157.89	16.11	-89.80	171.56	+100
Volta	440.78	167.67	-61.96	480.22	+100
Western	397.37	183.13	-53.91	435.32	+100
Western North	130.75	56.25	-56.98	154	+100

Admissions

According to the data presented in Table 3, the strike impacted negatively on hospital admissions. The average number of patients on admissions before and after the strike was more than twice the average number during the strike period. The percentage decrease in the mean admissions ranges from 46.42% (Bono region) to 94.58% (Upper West region). All the regions registered an increase in admissions, with corresponding a percentage increase ranging 77.13% (Central region) to +100% (e.g., Greater Accra).

Table 3: *Percentage change in the mean admissions during and after strike*

**Region**	**Before Strike**	**During Strike**		**After Strike**	
	Mean Admission	Mean Admission	% Δ from Before Strike	Mean Admission	% Δ from During Strike
Ahafo	71	5	-92.96	73	+100
Ashanti	57.5	26	-54.78	46.67	+79.5
Bono	116.33	62.33	-46.42	110	+76.48
Bono East	109.67	30.67	-72.03	101.67	+100
Central	81.71	36.86	-54.89	65.29	+77.13
Eastern	52.2	11.2	-78.54	54.8	+100
Greater Accra	50.17	11.83	-76.42	52.67	+100
North East	167.5	4	-97.61	189	+100
Northern	86	6	-93.02	129.14	+100
Oti	88.75	35	-60.56	77.25	+100
Savannah	42	20.5	-51.19	54.5	+100
Upper East	89	14.88	-83.28	77.88	+100
Upper West	75.78	4.11	-94.58	67.33	+100
Volta	120.33	32.11	-73.31	105.33	+100
Western	117.5	23.63	-79.89	119.38	+100
Western North	62.5	15.25	-75.60	66	+100

Deliveries Conducted

Table 4 showed that during the strike period, the average number of deliveries conducted across the regions was very low compared to the average number of deliveries conducted prior to the strike and after the strike. In Northern region, for example, an average of 25 deliveries were conducted prior to the strike. This reduced to 2 during the strike period but increased astronomically to 40 after the strike was called off In terms of percentage change in deliveries conducted, Savannah region reported the lowest decrease (10%), whereas Bono East reported the highest decrease (93.61%). Across the regions, there was an increase in deliveries conducted following the suspension of the strike action. With the exception of Greater Accra, all the other regions recorded more than 100% increase in mean deliveries after the strike was called relative to the strike period.

Table 4: *Percentage change in the mean deliveries conducted during and after strike*

**Region**	**Before Strike**	**During Strike**		**After Strike**	
	Mean Delivery	Mean Delivery	% Δ from Before Strike	Mean Delivery	% Δ from During Strike
Ahafo	18	3	-83.33	25	+100
Ashanti	12.33	1.45	-88.24	10.67	+100
Bono	17.83	6.17	-65.40	16.33	+100
Bono East	11	0.67	-93.91	12.67	+100
Central	12.43	3.43	-72.40	10.29	+100
Eastern	14.4	3.4	-76.39	12.8	+100
Greater Accra	22.17	8.83	-60.17	16.83	90.60
North East	19.5	4.5	-76.92	20	+100
Northern	25.71	2	-92.22	40	+100
Oti	9.5	6.25	-34.21	14	+100
Savannah	5	4.5	-10	12	+100
Upper East	38.5	5.38	-86.03	17	+100
Upper West	16.44	5	-69.59	17	+100
Volta	12.33	3	-75.67	11.22	+100
Western	12.13	5.63	-53.59	13	+100
Western North	7.75	1.25	-83.87	9	+100

Surgeries Performed

The data presented in Table 5 revealed that, the average number of surgeries performed decreased across the regions. In particular, the percentage decrease in mean surgeries ranges from 67.27% (Savannah region) to 100% (e.g., North East). As can be seen in Table 5 and Figure 4, no surgeries were performed in Ahafo and North East regions during the strike period. Whereas there was an increase in surgeries performed in most regions when the strike was called off relative to the strike period (≥ 100% percentage increase in mean), only Savannah region recorded a decrease, corresponding to a percentage of 55.56%.

Table 5: *Percentage change in the mean surgeries performed during and after strike*

**Region**	**Before Strike**	**During Strike**		**After Strike**	
	Mean Surgeries	Mean Surgeries	% Δ from Before Strike	Mean Surgeries	% Δ from During Strike
Ahafo	1	0	-100	2	100
Ashanti	6.25	1.75	-72	5.42	+100
Bono	18.33	4.17	-77.25	19.17	+100
Bono East	17.33	2.33	-86.56	16	+100
Central	7.14	1.29	-81.93	6.57	+100
Eastern	18	1.4	-92.22	23.2	+100
Greater Accra	12.67	3.33	-73.71	42.5	+100
North East	5	0	-100	2.5	100
Northern	8.57	0.86	-89.97	12.43	+100
Oti	5.25	1.5	-71.43	4.75	+100
Savannah	27.5	9	-67.27	4	-55.56
Upper East	7.25	0.5	-93.10	12	+100
Upper West	6.22	1.22	-80.39	6.33	+100
Volta	8.33	0.89	-89.32	8.56	+100
Western	10.75	2.75	-74.42	10.25	+100
Western North	3.75	0.75	-80	5.5	+100

Reproductive Health Services

Data on reproductive health services used before, during and after the strike were summarized in Table 6. Across the regions, the results showed that the average number of people who accessed reproductive health services dwindled dramatically during the strike period, compared with the periods before and after strike. In three of the regions (i.e., Ahafo, Central and Northern), no one accessed reproductive health services during the strike period. More importantly, the corresponding percentage decrease in mean reproductive service use during strike, relative to before the strike ranges from 45.74% (Bono region) to 99.80% (Volta region). Likewise, the regions experienced a significant increase (i.e., ≥ 60.74 %) in reproductive service use following the suspension of the strike. In Savannah region, however, the results showed that service used decreased after the strike was called off, compared with during the strike, corresponding to a 96% decrease.

Table 6: *Percentage change in the mean reproductive service use (RSU) during and after strike*

**Region**	**Before Strike**	**During Strike**		**After Strike**	
	Mean RSU	Mean RSU	% Δ from Before Strike	Mean RSU	% Δ from During Strike
Ahafo	68	0	-100	230	100
Ashanti	88.5	8.92	-89.92	119.5	+100
Bono	43	23.33	-45.74	37.5	60.74
Bono East	63.67	5	-92.15	48.67	+100
Central	66.5	0	-100	55.86	100
Eastern	21.6	0.4	-98.15	59.8	+100
Greater Accra	78.83	13.5	-82.87	76.17	+100
North East	107	0	-100	161.5	100
Northern	38.33	0	-100	60.33	100
Oti	27	1	-96.30	37.75	+100
Savannah	14	25	78.57	1	-96
Upper East	51.75	6	-88.41	55.38	+100
Upper West	33.56	1.11	-96.69	31.89	+100
Volta	77.78	0.11	-99.86	66.56	+100
Western	58.25	2.25	-96.14	65	+100
Western North	59.5	16.25	-72.69	53.25	+100

Antenatal Services

The data provided in Table 7 represented the average number of antenatal service users at hospitals located across the regions before, during, and after strike. Similar to the trend reported above, the average number of people who utilized reproductive health services decreased greatly during the period of strike, compared with the period before and after the strike. Ashanti region recorded the least percentage decrease in mean antenatal use (76.13%), whereas North East and Northern regions registered the highest decrease (100%). Relatedly, there was more than 100% increase in antenatal service use following the suspension of the strike action, relative to during the strike, across all the regions.

Table 7: Percentage change in the mean antenatal service use (ASU) during and after strike

**Region**	**Before Strike**	**During Strike**		**After Strike**	
	Mean ASU	Mean ASU	% Δ from Before Strike	Mean ASU	% Δ from During Strike
Ahafo	135	9	-93.33	175	+100
Ashanti	68.42	16.33	-76.13	79.67	+100
Bono	89	13.17	-85.20	115.17	+100
Bono East	215	67.67	-68.53	234.33	+100
Central	58.43	6.57	-88.76	70.43	+100
Eastern	63.2	2.2	-96.52	124.6	+100
Greater Accra	87.83	19.67	-77.60	98	+100
North East	150	0	-100	187	+100
Northern	164	0	-100	291.71	+100
Oti	60	16.75	-72.08	70.75	+100
Savannah	41.5	4.5	-89.16	37.5	+100
Upper East	27	0.5	-98.15	30.25	+100
Upper West	29.89	0.78	-97.39	39.22	+100
Volta	85.33	0.75	-99.12	105.67	+100
Western	104.5	24.88	-76.19	117.5	+100
Western North	34.5	1	-97.10	48	+100

Access to Health Services at Polyclinics during Strike

The impacts of the strike action on access to health services at the polyclinics are summarized below;

Outpatient Department Attendance

The data on OPD attendance in Table 8 and Figure 7 revealed that the average number of people accessing OPD services reduced drastically during the strike period, compared with before strike. The percentage decrease in OPD attendance ranges from 23.44% (Central region) to 100% in Bono region. Taking Ashanti region as example, before the strike, 299 people accessed OPD services. However, this dropped to 55 during the strike period, representing 81.61% decrease. The results further showed that, OPD attendance increased above 100% across the regions when the strike was called off.

Table 8: *Percentage change in outpatient department attendance during and after strike*

**Region**	**Before Strike**	**During Strike**		**After Strike**	
	OPD attendance	OPD Attendance	% Δ from Before Strike	OPD Attendance	% Δ from During Strike
Ashanti	299	55	-81.61	409	+100
Bono	102	0	-100	110	+100
Central	128	98	-23.44	133	+100
Greater Accra*	66	7.5	-88.64	38.5	+100
North East	142	0	-100	144	+100
Upper West	65	8	-87.69	62	+100
Western North	17	24	41.18	14	+100

**= mean/average data*

Admissions

As shown in Table 9, the average number of patients on admissions declined during the strike period across the regions. Illustrating with Central region, the average number of patients on admission during the strike was 12, compared with 20 before and 7 after the strike. In Bono, Greater Accra and North East regions, there were zero admissions during the strike period. All the regions witnessed an increase in admissions after the strike, ranging from 41.67% to +100%.

Table 9: *Percentage change in mean admissions during and after strike*

**Region**	**Before Strike**	**During Strike**		**After Strike**	
	Admissions Data	Admissions Data	% Δ from Before Strike	Admissions Data	% Δ from During Strike
Ashanti	29	10	-65.52	42	+100
Bono	4	0	-100	9	+100
Central	20	12	-40	7	41.67
Greater Accra*	13	0	-100	4	+100
North East	18	0	-100	2	+100

**= mean/average data*

Deliveries Conducted

Access to delivery services was also negatively affected during the strike period, as shown in Table 10 and Figure 9. The average number of deliveries conducted reduced during the strike period, compared with the period before and after the strike. The corresponding percentage decrease ranges from 25% (Central region) to 100% (e.g., Bono region). In Ashanti region, for instance, 2 deliveries were conducted on average during the strike, whereas the average number of deliveries before and after the strike stood at 5 and 15, respectively. In regions such as Bono and North East, there were no deliveries performed during the strike period. However, all the regions recorded more than 100% increase in deliveries conducted after the strike was called off.

Table 10: *Percentage change in deliveries conducted during and after strike*

**Region**	**Before Strike**	**During Strike**		**After Strike**	
	Delivery Data	Delivery Data	% Δ from Before Strike	Delivery Data	% Δ from During Strike
Ashanti	5	2	-60	15	+100
Bono	5	0	-100	4	+100
Central	4	3	-25	8	+100
Greater Accra*	1.5	0.5	-66.67	0	+100
North East	8	0	-100	5	+100
Upper West	3	2	-33.33	4	+100

**= mean/average data*

Reproductive Health Services

The result in Table 11 showed that, during the strike, no reproductive services were provided at Ashanti, Bono, Greater Accra and Upper West regions. Only Central region provided reproductive health services during the strike period. In Ashanti and Bono regions, more reproductive services were provided after the strike was called off, compared with before the strike was declared.

Table 11: *Percentage change in reproductive service use (RSU) during and after strike*

**Region**	**Before Strike**	**During Strike**		**After Strike**	
	RSU Data	RSU Data	% Δ from Before Strike	RSU Data	% Δ from During Strike
Ashanti	256	0	-100	251	+100
Bono	8	0	-100	12	+100
Central	2	5	+100	4	-20
Greater Accra*	40	0	-100	37	+100
Upper West	3	0	-100	2	+100

**= mean/average data*

Antenatal Services

The data on the use of antenatal services were summarized in Table 12. In Ashanti, Bono and Greater Accra regions, there was no antenatal service use during the strike period. Antenatal service during the strike was reported in the Central region where the average number of service users during the strike period was more than before and after the strike. This was followed by

Upper West, except that the average number of service users during the strike was less than before and after the strike.

Table 12: Percentage change in antenatal service use (RSU) during and after strike

**Region**	**Before Strike**	**During Strike**		**After Strike**	
	ASU Data	ASU Data	% Δ from Before Strike	ASU Data	% Δ from During Strike
Ashanti	146	0	-100	143	+100
Bono	39	0	-100	59	+100
Central	27	35	-29.63	21	-40
Greater Accra*	63.5	0	-100	41	+100
Upper West	6	2	-66.67	3	50

**= mean/average data*

Access to Health Services at Health Centers during Strike

Outpatient Department Attendance

As shown in Table 13, the average OPD attendance at health centers across the regions decreased during the strike period. Three of regions (e.g., Greater Accra, Northern and Western regions) recorded no OPD attendance during the strike. For the remaining regions, percentage decrease in average OPD attendance ranges from 46.86% (Western North region) to 94.49% (Upper West). In the same vein, there was over 100% increase in the average OPD when the strike was called off.

Table 13: Percentage change in the mean OPD attendance during and after strike

**Region**	**Before Strike**	**During Strike**		**After Strike**	
	Mean OPD Attendance	Mean OPD Attendance	% Δ from Before Strike	Mean OPD Attendance	% Δ from During Strike
Ashanti	44.25	11.08	-74.96	48.92	+100
Bono	66	12	-81.82	132	+100
Central	53.62	5.92	-88.96	55.23	+100
Greater Accra	36	0	-100	31	+100
North East	105.33	33	-68.67	111.33	+100
Northern	16	0	-100	12	+100
Savannah	10.83	3	-72.30	12.17	+100
Upper East	40.5	2.75	-93.21	37.75	+100
Upper West	31.75	1.75	-94.49	24.25	+100
Volta	82	14.5	-82.32	94.5	+100
Western	18.67	0	-100	26	+100
Western North	28.6	15.2	-46.85	36.2	+100

Deliveries Conducted

Table 14 showed that the average deliveries conducted in the health centers reduced significantly during the strike period. More specifically, the percentage decrease ranges from 14.29% (Western North) to 100% (e.g., Northern region). Following the suspension of the strike, average deliveries conducted increased beyond 100% from during the strike period all but one region (Northern region) where there was deliveries conducted.

Table 14: *Percentage change in the mean deliveries conducted during and after strike*

Region	Before Strike	During Strike		After Strike	
	Mean Deliveries	Mean Deliveries	% Δ from Before Strike	Mean Deliveries	% Δ from During Strike
Ashanti	2.75	0.58	-78.91	2.58	+100
Bono	0.5	0	-100	1	100
Central	1.69	0.67	-60.36	2.69	+100
Greater Accra	1	0.5	-50	4	+100
North East	0.83	0.17	-79.52	1	100
Northern	1	0	-100	0	0
Savannah	1.17	1.5	28.21	1	+100
Upper East	1.75	0.75	-57.14	1.5	+75
Upper West	0.5	0.38	-24	0.75	+100
Volta	2	0	-100	3.5	+100
Western	1.33	0	-100	0.33	+100
Western North	1.4	1.2	-14.29	1.6	+33

Discusion

Nurses and midwives are extremely important in the provision of healthcare services in Ghana, as stated previously. The health system is functional and operational due to the contributions of nurses and midwives. As a result, the strike action embarked on by the Ghana Registered Nurses and Midwives Association on 21st septermber 2020 had a significant impact on access to health care services.

Consistent with the existing literature (Aro & Hosia, 1987; Njuguna, 2015; Scanlon et al., 2021; Sievertsen & Wüst 2016), the results of the study showed that access to healthcare services decreased significantly across the regions and health facilities during the strike period. For example, the average numbers of patients on admission before and after the strike were more than twice the average number during the strike period. The reduction in healthcare deliveries was not

only evident from the number of OPD attendance, admissions, deliveries and surgeries but was also evident in the number reproductive and child health used during the strike action. Across the regions, there was a significant reduction in reproductive service utilization. In three of the regions (i.e., Ahafo, Central and Northern), no one accessed reproductive health services during the strike period. The average number of people who utilized antenatal services decreased greatly during the period of strike, compared with the period before and after the strike. In the North East and Northern regions, there was a zero use of antenatal services during the strike.

The decrease could be attributed to the fact that nurses and midwives are "basic unit" of the healthcare professionals such that in their absence, the general public is not able to initiate help-seeking behavior from health professionals. Indeed, nurses activate the healthcare system through the provision of basic health services such as temperature, blood pressure and blood glucose checks that form the foundation for advanced and detailed healthcare services. Besides, nurses provide major support to other health professionals in rendering healthcare services. Surgeries cannot be performed by only surgeons without the professional skills of nurses. All surgical cases require admissions before and after the surgical procedure. Without adequate number of nurses to care for patients before and after surgery, mortalities are inevitable. Therefore, the absence of nurses tend to create a significant professional gap that other health professionals, by virtue of their training and specialization, are not able to immediately and appropriately fulfill their roles. Because the general public is aware of the duties and responsibilities nurses and midwives play in healthcare delivery, news about strike by these professionals could affect an individual's decision to seek professional care from the striking hospitals.

Although data were not obtained on service users, however, previous studies have shown that social and economically disadvantaged groups are extremely vulnerable to the negative

repercussions of strike as they are unable to pay for services in a private hospital (Irimu et al., 2018). These individuals may resort to the use of herbal preparations and self-medication that often exacerbate their conditions, culminating into increased morbidity and mortality (Irimu et al., 2018; Oleribe et al., 2018; Waithaka et al., 2020). In another point of discussion, the increase in healthcare access post-strike reflects an increase in unmet needs of the public (Aro & Hosia, 1987). The unmet health needs will be compounded should the strike persi[illegible]

Limitations of the Study

The health facilities were not randomly selected for inclusion in this study, thereby limiting the generalization of the findings reported in this study. Given the nature of research design, particularly data collection method and data collected, it was impossible to determine whether and to what extent other factors beyond the strike action contributed to the decrease in access to healthcare during strike period and an increase post-strike.

Conclusions

The strike action by the nurses and midwives in Ghana significantly reduced access to a range of health services, including reproductive health services, deliveries and antenatal services. The distribution of the negative consequences across the regions appear similar. It is evident that strike action by nurses and midwives or other health professionals will derail the efforts made by the Government of Ghana and development partners to boost healthcare delivery and access in Ghana. It is also imperative to state that the attainment of the goal 3 (health and well-being) of SDG in Ghana will be negatively affected with strike action by nurses and midwives. Therefore, efforts should be made by the Government of Ghana to address the needs of nurses and midwives to avert future strike actions.

References

Agyepong, I. A., Kodua, A., Adjei, S., & Adam, T. (2012). When 'solutions of yesterday become problems of today': crisis-ridden decision making in a complex adaptive system (CAS)—the Additional Duty Hours Allowance in Ghana. *Health Policy and Planning, 27*(suppl_4), iv20-iv31.

Aro, S., & Hosia, P. (1987). Effects of a doctors' strike on primary care utilization in Varkaus, Finland. *Scandinavian Journal of Primary Health Care, 5*(4), 245-251.

Aturaka, S. O., Paul, A. O., Amosu, A., Sanni, F., Orenyi, M., Dakwat, M., . . . Chiegil, R. J. Effect of Health Workers Strikes on Quality of Care in Health Institution in Cross River State, Nigeria.

Bhuiyan, M., & Machowski, A. (2012). Impact of 20-day strike in Polokwane hospital (18 August-6 September 2010). *South African Medical Journal, 102*(9), 755-756.

Furnivall, D., Bottle, A., & Aylin, P. (2018). Retrospective analysis of the national impact of industrial action by English junior doctors in 2016. *BMJ Open, 8*(1), e019319.

Gyamfi, G. D. (2011). Assessing the effects of industrial unrest on Ghana health service: A case study of nurses at Korle-Bu teaching hospital. *International Journal of Nursing and Midwifery, 3*(1), 1-5.

Irimu, G., Ogero, M., Mbevi, G., Kariuki, C., Gathara, D., Akech, S., . . . English, M. (2018). Tackling health professionals' strikes: an essential part of health system strengthening in Kenya. *BMJ Global Health, 3*(6).

Manthous, C. A. (2014). Labor unions in medicine: the intersection of patient advocacy and self-advocacy. *Medical Care, 52*(5), 387-392.

Njuguna, J. (2015). Impact of health workers' strike in August 2014 on health services in Mombasa County Referral Hospital, Kenya. *Journal of Health Care for the Poor and Underserved, 26*(4), 1200-1206.

Njuguna, J. (2018). Impact of Nurses' Strike in Kenya on Number of Fully Immunized Infants in 18 County Referral Hospitals. *Journal of Health Care for the Poor and Underserved, 29*(4), 1281-1287.

Oleribe, O. O., Ezieme, I. P., Oladipo, O., Akinola, E. P., Udofia, D., & Taylor-Robinson, S. D. (2016). Industrial action by healthcare workers in Nigeria in 2013–2015: an inquiry into causes, consequences and control—a cross-sectional descriptive study. *Human Resources for Health, 14*(1), 1-10.

Oleribe, O. O., Udofia, D., Oladipo, O., Ishola, T. A., & Taylor-Robinson, S. D. (2018). Healthcare workers' industrial action in Nigeria: a cross-sectional survey of Nigerian physicians. *Human Resources for Health, 16*(1), 1-10.

Ruiz, M., Bottle, A., & Aylin, P. (2013). A retrospective study of the impact of the doctors' strike in England on 21 June 2012. *Journal of the Royal Society of Medicine, 106*(9), 362-369.

Scanlon, M. L., Maldonado, L. Y., Ikemeri, J. E., Jumah, A., Anusu, G., Bone, J. N., . . . Songok, J. (2021). A Retrospective Study of the Impact of Health Worker Strikes on Maternal and Child Health Care Utilization in Western Kenya.

Sheahan, K. H., Kennedy, A. G., Tompkins, B. J., Repp, A. B., & Gilbert, M. P. (2021). The Impact of a Nursing Strike on Glycemic Control in Hospitalized Patients with Diabetes. *Cureus, 13*(6).

Van Rensburg, A. J., & van Rensburg, D. J. (2013). Nurses, industrial action and ethics: considerations from the 2010 South African public-sector strike. *Nursing Ethics, 20*(7), 819-837.

Waithaka, D., Kagwanja, N., Nzinga, J., Tsofa, B., Leli, H., Mataza, C., . . . Barasa, E. (2020). Prolonged health worker strikes in Kenya-perspectives and experiences of frontline health managers and local communities in Kilifi County. *International journal for equity in health, 19*(1), 23.
