# Supplementary material for: The impact of strike action by Ghana registered nurses and midwives on the access to and utilization of healthcare services
Source: PLoS One. 2022 Oct 14;17(10):e0275661. doi: 10.1371/journal.pone.0275661 (PMC9565728; doi:10.1371/journal.pone.0275661)
Supplement: Attachment — Submitted filename: PONE-D-21-35187_R1__.pdf [file pone.0275661.s004.pdf]

PLOS ONE

# A Multi-Level Impact Analysis of Strike Action by Ghana Registered Nurses and Midwives on Access to and Utilization of Healthcare Services.

--Manuscript Draft--

| | |
|---|---|
| **Manuscript Number:** | PONE-D-21-35187R1 |
| **Article Type:** | Research Article |
| **Full Title:** | A Multi-Level Impact Analysis of Strike Action by Ghana Registered Nurses and Midwives on Access to and Utilization of Healthcare Services. |
| **Short Title:** | Impact of Strike Action by Ghana Registered Nurses and Midwives |
| **Corresponding Author:** | Samuel Adjorlolo<br>University of Ghana<br>Accra, GHANA |
| **Keywords:** | Strike, nurses and midwives, healthcare access and use, Ghana |
| **Abstract:** | Background : As the largest professional group, nurses and midwives play instrumental roles in healthcare delivery, supporting the smooth administration and operation of the health system. Consequently, the withdrawal of nursing and midwifery services via strike action has direct and indirect detrimental effects on access to healthcare.<br>Objective : The current study examined the impact of strike action by nurses and midwives with respect to access to and utilization of health services.<br>Method: Data were collected retrospectively from a total of 181 health facilities from all the 16 administrative regions of Ghana, with the support of field officers. Because the strike lasted for 3 days, the data collection span three consecutive days before the strike, three days of the strike and three consecutive days after the strike. Data analysis was focused comparing the utilization of healthcare services before, during and after strike. Data were analysed and presented on the various healthcare services. This was done separately for the health facility type and the 16 administrative regions.<br>Findings : The results showed that; (1) the average number of patients or clients who accessed healthcare services reduced drastically during the strike period, compared with before the strike. Majority of the regions recorded more than 70% decrease in service use during the strike period; (2) the average number of patients or clients who accessed healthcare services after the strike increased by more than 100% across majority of the regions.<br>Conclusion : The study showed that strike action by nurses and midwives negatively affected access to and utilization of healthcare services. |
| **Order of Authors:** | Samuel Adjorlolo |
| | Perpetual Ofori Ampofo |
| | Margaretta Gloria Chandi |
| | Francis Kwaku Wuni |
| | Ernestina Asiedu |
| | Vida Ami Kukula |
| | Sampson Opoku |
| | David Tenkorang-Twum |
| **Response to Reviewers:** | Find attached the document named "Response to Reviewer Comments" |
| **Additional Information:** | |
| **Question** | **Response** |
| **Financial Disclosure**<br><br>Enter a financial disclosure statement that | Not applicable |

Powered by Editorial Manager® and ProduXion Manager® from Aries Systems Corporation

| | |
|---|---|
| describes the sources of funding for the work included in this submission. Review the submission guidelines for detailed requirements. View published research articles from *PLOS ONE* for specific examples.<br><br>This statement is required for submission and **will appear in the published article** if the submission is accepted. Please make sure it is accurate.<br><br>**Unfunded studies**<br>Enter: *The author(s) received no specific funding for this work.*<br><br>**Funded studies**<br>Enter a statement with the following details:<br>• Initials of the authors who received each award<br>• Grant numbers awarded to each author<br>• The full name of each funder<br>• URL of each funder website<br>• Did the sponsors or funders play any role in the study design, data collection and analysis, decision to publish, or preparation of the manuscript?<br>• **NO** - Include this sentence at the end of your statement: *The funders had no role in study design, data collection and analysis, decision to publish, or preparation of the manuscript.*<br>• **YES** - Specify the role(s) played.<br><br>* typeset | |
| **Competing Interests**<br><br>Use the instructions below to enter a competing interest statement for this submission. On behalf of all authors, disclose any competing interests that could be perceived to bias this work—acknowledging all financial support and any other relevant financial or non-financial competing interests.<br><br>This statement is required for submission and **will appear in the published article** if the submission is accepted. Please make sure it is accurate and that any funding | None has been declared |

| | |
|---|---|
| sources listed in your Funding Information later in the submission form are also declared in your Financial Disclosure statement.<br><br>View published research articles from *PLOS ONE* for specific examples.<br><br>**NO authors have competing interests**<br><br>Enter: *The authors have declared that no competing interests exist.*<br><br>**Authors with competing interests**<br><br>Enter competing interest details beginning with this statement:<br><br>*I have read the journal's policy and the authors of this manuscript have the following competing interests: [insert competing interests here]*<br><br>* typeset | |
| **Ethics Statement**<br><br>Enter an ethics statement for this submission. This statement is required if the study involved:<br><br>• Human participants<br>• Human specimens or tissue<br>• Vertebrate animals or cephalopods<br>• Vertebrate embryos or tissues<br>• Field research<br><br>Write "N/A" if the submission does not require an ethics statement.<br><br>General guidance is provided below. Consult the submission guidelines for detailed instructions. **Make sure that all information entered here is included in the Methods section of the manuscript.** | This is a retrospective study in which data on the number of people who utilized various healthcare services before, during and after strike action by nurses and midwives was gathered. The study did not involve direct human subject engagement. Rather, data were obtained from institutional archives as an aggregate data. The focus was on how many people visited or utilized healthcare services within the frame above without focusing on the background or demographics of users of healthcare services. The data collected were also devoid of identifying information relating to the facilities. This means that neither the facility nor clients/patients will be identified. Thus, data were fully anonymized. The data on number of people accessing healthcare services is notably a public data in Ghana. Given the nature of the study, we did not seek IRB approval. The project was underpinned by other relevant ethical considerations in research, including confidentiality, data safety and data protection. The field officers volunteered to be part of the project, despite being recommended by their regional executives. Indeed, out of the 150 persons recommended as field officers, only 112 agreed to take part in the project. The rest withdrew from the study without suffering any consequences. Access to data was restricted to the research team or other individuals supporting the project, mainly data analysts. These individuals signed a statement confirming that they would adhere to the study procedures regarding confidentiality. The data collected were analyzed as regional aggregate data to further delink the facilities from the results. The compensation for the field officers ranges from GH¢100 to GH¢200 and was proportional to the number of facilities each field officer obtained data from. The compensation was to be used for transportation, communication and other costs relating to the project. |

Powered by Editorial Manager® and ProduXion Manager® from Aries Systems Corporation

| | |
|---|---|
| **Format for specific study types**<br><br>**Human Subject Research (involving human participants and/or tissue)**<br>• Give the name of the institutional review board or ethics committee that approved the study<br>• Include the approval number and/or a statement indicating approval of this research<br>• Indicate the form of consent obtained (written/oral) or the reason that consent was not obtained (e.g. the data were analyzed anonymously)<br><br>**Animal Research (involving vertebrate animals, embryos or tissues)**<br>• Provide the name of the Institutional Animal Care and Use Committee (IACUC) or other relevant ethics board that reviewed the study protocol, and indicate whether they approved this research or granted a formal waiver of ethical approval<br>• Include an approval number if one was obtained<br>• If the study involved *non-human primates*, add *additional details* about animal welfare and steps taken to ameliorate suffering<br>• If anesthesia, euthanasia, or any kind of animal sacrifice is part of the study, include briefly which substances and/or methods were applied<br><br>**Field Research**<br><br>Include the following details if this study involves the collection of plant, animal, or other materials from a natural setting:<br>• Field permit number<br>• Name of the institution or relevant body that granted permission | |
| **Data Availability**<br><br>Authors are required to make all data underlying the findings described fully available, without restriction, and from the time of publication. PLOS allows rare exceptions to address legal and ethical concerns. See the PLOS Data Policy and FAQ for detailed information. | Yes - all data are fully available without restriction |

| | |
|---|---|
| A Data Availability Statement describing where the data can be found is required at submission. Your answers to this question constitute the Data Availability Statement and **will be published in the article**, if accepted.<br><br>**Important:** Stating 'data available on request from the author' is not sufficient. If your data are only available upon request, select 'No' for the first question and explain your exceptional situation in the text box.<br><br>Do the authors confirm that all data underlying the findings described in their manuscript are fully available without restriction? | |
| **Describe where the data may be found in full sentences. If you are copying our sample text, replace any instances of XXX with the appropriate details.**<br><br>• If the data are **held or will be held in a public repository**, include URLs, accession numbers or DOIs. If this information will only be available after acceptance, indicate this by ticking the box below. For example: *All XXX files are available from the XXX database (accession number(s) XXX, XXX.*).<br>• If the data are all contained **within the manuscript and/or Supporting Information files**, enter the following: *All relevant data are within the manuscript and its Supporting Information files.*<br>• If neither of these applies but you are able to provide **details of access elsewhere**, with or without limitations, please do so. For example:<br><br>*Data cannot be shared publicly because of [XXX]. Data are available from the XXX Institutional Data Access / Ethics Committee (contact via XXX) for researchers who meet the criteria for access to confidential data.*<br><br>*The data underlying the results presented in the study are available from (include the name of the third party* | All relevant data are within the manuscript and its Supporting Information files |

| | |
|---|---|
| *and contact information or URL).*<br>• This text is appropriate if the data are owned by a third party and authors do not have permission to share the data.<br><br>* typeset | |
| Additional data availability information: | |

Powered by Editorial Manager® and ProduXion Manager® from Aries Systems Corporation

Manuscript Click here to access/download;Manuscript;Manuscript.docx

# A Multi-Level Impact Analysis of Strike Action by Ghana Registered Nurses and Midwives on Access to and Utilization of Healthcare Services

Perpetual Ofori Ampofo[1]; David Tenkorang-Twum[1,2]; Samuel Adjorlolo[3,4]; Margaretta Gloria Chandi[5]; Francis KwakuWuni[6], Ernestina Asiedu[7], Vida Ami Kukula[8], Sampson Opoku[9]

1. Ghana Registered Nurses and Midwives' Association of Ghana. Head Office, Okponglo, Accra, Ghana.

2. Department of Adult Health, School of Nursing and Midwifery, University of Ghana, Legon, Accra, Ghana.

3. Department of Mental Health, School of Nursing and Midwifery, University of Ghana, Legon, Accra, Ghana.

4. Research and Grant Institute of Ghana, P. Box LG 1004, Legon Post Office, Accra, Ghana.

5. GA West Municipal health directorate, P. O .Box AM187, Accra, Ghana.

6. Regional hospital, P.O. Box 26, Bolgatanga.

7. Department of Child and Maternal Health, School of Nursing and Midwifery, University of Ghana, Accra, Ghana.

8. Dodowa Health Research Center, Dodowa, Accra, Ghana.

9. P. O. Box KF 735. Koforidua, Eastern Region, Ghana.

**Corresponding Author**

Dr. Samuel Adjorlolo, Department of Mental Health, School of Nursing and Midwifery, College of Health Sciences, University of Ghana, P. O. Box LG 43, Legon, Accra, Ghana; email: sadjorlolo@ug.edu.gh

**A Multi-Level Impact Analysis of Strike Action by Ghana Registered Nurses and Midwives on Access to and Utilization of Healthcare Services**


## Abstract

**Background**: As the largest professional group, nurses and midwives play instrumental roles in healthcare delivery, supporting the smooth administration and operation of the health system. Consequently, the withdrawal of nursing and midwifery services via strike action has direct and indirect detrimental effects on access to healthcare.

**Objective**: The current study examined the impact of strike action by nurses and midwives with respect to access to and utilization of health services.

**Method:** Data were collected retrospectively from a total of 181 health facilities from all the 16 administrative regions of Ghana, with the support of field officers. Because the strike lasted for 3 days, the data collection span three consecutive days before the strike, three days of the strike and three consecutive days after the strike. Data analysis was focused comparing the utilization of healthcare services before, during and after strike. Data were analysed and presented on the various healthcare services. This was done separately for the health facility type and the 16 administrative regions.

**Findings**: The results showed that; (1) the average number of patients or clients who accessed healthcare services reduced drastically during the strike period, compared with before the strike. Majority of the regions recorded more than 70% decrease in service use during the strike period; (2) the average number of patients or clients who accessed healthcare services after the strike increased by more than 100% across majority of the regions.

**Conclusion**: The study showed that strike action by nurses and midwives negatively affected access to and utilization of healthcare services.



**Keywords**: Strike, nurses and midwives, healthcare access and use, Ghana

## Introduction

The services provided by nurses and midwives are essential in the attainment of universal healthcare coverage; hence the withdrawal of such services via strike action has direct and indirect detrimental effects (Van Rensburg & van Rensburg, 2013). The current study examines the impact of strike action declared and undertaken by members of Ghana Registered Nurses and Midwives Association (GRNMA), the umbrella body of nurses and midwives in Ghana. Strike is the last weapon used by employees to fight for salary and improved working conditions. Employees who feel their rights and wellbeing have been violated, interfered with, or disregarded by their employer may decide to strike as the only practical way to get their complaints acknowledged and addressed (Osakede, 2014). Strike actions by healthcare workers take varying forms such as suspension of general healthcare delivery services, provision of only emergency services, cessation of work for few hours, days, weeks and months (Agyepong et al., 2012; Manthous, 2014).

The decision to embark on strike is influenced by a myriad of factors, predominant among them is conditions of service. Illustrating, among Ghanaian nurses, dissatisfaction with working conditions, particularly salary, was cited as the motivating factor for their strike actions. More specifically, the nurses expressed concern about the large wage disparity in reference to the medical doctors, rather than equal pay (Gyamfi, 2011). The connection between remuneration of health workers and the emergence of strike actions is further supported by a study in Nigeria. According to Oleribe et al., (2016), the majority of strikes in Nigeria are primarily motivated by a desire for increased pay. In Tanzania, the request for improved working conditions in the areas of infrastructure, medications, equipment, and other medical supplies, as well as underpayment of salary and allowances were the reasons for doctors’ strike in 2012 (Isangula, 2012). Strike actions by healthcare workers in Egypt in 2011 was intended to persuade authorities to reinforce safety in

healthcare facilities, increase healthcare budget, and improve on the deteriorating state of facilities in the country's  health care system(Shukla, 2012).

Other factors associated with strike actions include: poor working environment, demotivation among staff, delayed or unfairness in promotions (Gyamfi, 2011; Oleribe et al., 2016; Waithaka et al., 2020). Dissatisfaction with leadership and management has also been implicated in strike action by healthcare workers in Nigeria (Oleribe et al., 2016). Notable among the managerial inefficiencies include delayed promotions and limited educational and training support (Oleribe et al., 2018).  Other studies have registered solidarity with a colleague for an unlawful assault, or dismissal as reasons why health workers may embark on strike action (Oleribe et al., 2018; Waithaka et al., 2020).

Restricted access to healthcare, occasioned by strike, is a recipe for increased mortality and morbidity, decreased revenue mobilization and other undesirable outcomes. In Nigeria, strike action by healthcare workers potentiated increased referrals to private health facilities (66.0%) and its resultant challenges such as substandard treatment and high cost of services (Oleribe et al., 2016). In a related study in Kenya involving nurses, Njuguna (2015) reported that the strike action had a negative impact on vaccination services for children. For example, there was a 56.9% decrease in the number of newborns who received vaccinations during the strike in public health facilities. However, the proportion of immunizations in Faith –based hospitals increased by 252%. Scanlon et al. (2021) also established a robust link between strike action by healthcare workers and poor maternity and health care utilization, including ANC and delivery at a health facility among pregnant women, and delays in a child's first oral polio vaccination in Kenya. In a related Kenyan study, outpatient attendance declined by 64.4%, special clinics attendance by 74.2%, deliveries by 53.5% and inpatient admissions by 57.8% when nurses and other healthcare

professionals embarked on strike (Njuguna, 2015). A decrease in health services utilization and admissions during the period of strike by nurses and medical doctors in Kenya contributed to a decrease in mortality rates registered across hospitals in Kenya (Kaguthi et al., 2020; Njuguna, 2015). Similar findings have been reported in high income countries, including US (Sheahan et al., 2021), England (Furnivall et al., 2018; Ruiz et al., 2013) and Finland (Aro & Hosia, 1987). For example, Ruiz et al. (2013) in their study in England found that compared with the non-strike period, emergency admissions fell by 2.4% while the elective admissions decreased by 12.8%. The authors reported a 7.8% drop in the number of outpatients seen by medical staff on the day of the strike and a 45.5% increase in the number of cancelled appointments by NHS hospitals, while accident and emergency attendances dropped by 4.7% (Ruiz et al., 2013).

The GRNMA, like other professional associations and labor unions, has embarked on several strike actions since 1978 as a broader strategy to obtain improved and better conditions of services for her members (Gyamfi, 2011). The most recent strike action embarked on by the GRNMA and allied health professionals recently was from September 21$^{st}$ to 23$^{rd}$, 2020 after negotiations with the Government (employer) for improved conditions of service yielded no results. As noted above, strike actions generally leads to a reduction in the number of people accessing healthcare services (Bhuiyan & Machowski, 2012; Oleribe et al., 2018). However, our understanding of the extent of disruption in access to a range of healthcare services is limited as majority of previous studies have tended to focus their attention on services rendered by a unit or department in hospital or health facility (exceptions see Njuguna, 2015). Ghana's healthcare delivery operates on three levels, thus primary, secondary, and tertiary. At the primary level, both preventive and promotive services are provided by Community health planning services (CHPS) centers, health centers, and polyclinics. They serve a population of approximately 20,000 people.

At the secondary level, curative services are provided by district hospitals and serve an average population of 100,000 to 200,000 people whiles at the tertiary level, curative and rehabilitative services are delivered at the regional/teaching hospitals which serve approximately an average population of about 1.2 million (Ghana Health Service[GHS], 2003). The healthcare delivery system has witnessed an increase in the number of healthcare professionals. For example, nurse-patient ratio has improved from 1:2,172 in 2013 to 1:505 in 2017 with the total number of nursing workforce increasing from 12,245 in 2013 to 58,608 in 2017. Again, the midwife to women in fertile age (WIFA) ratio has improved from 1:1533 to 1:704 with the total number of midwives increasing from 4,185 to 9884 (GHS, 2018). As the largest workforce in the healthcare system, nurses and midwives have significant roles to contribute to the attainment of universal health coverage (UHC). Therefore, understanding how strike actions by these professional groups impact the range of healthcare services available to the public will equip stakeholders to take appropriate steps to respond appropriately and timely to strike notices, avert strike actions to safeguard the health of the population. Second, although nurses and midwives and other health professionals in Ghana do embark on strike, there is no comprehensive and systematically generated evidence on the impact of their strike actions on access to healthcare. The lack of empirical data in this regard makes it extremely difficult for the nurses and midwives association (i.e., GRNMA) to quantify the contributions of her members to healthcare delivery and to make a compelling case for better conditions of service. At the same time, the employer is unable to readily envisage the devastation to access to healthcare delivery occasioned by the strike action of nurses and midwives and other health professionals. The current study fills this void by investigating the impact of the 2020 strike action by GRNMA and its Allied health professionals on access to a range of healthcare services in Ghana.

*Study Objectives*

The objectives of the study are as follows;

1. Determine the number of people accessing and utilization health services before, during and after strike action by nurses and midwives.
2. Compare the number of people who accessed essential healthcare services before, during and after the strike was called off.

## Method

*Project Setting*

Data were gathered from health facilities located across the 16 regions in Ghana and across all the levels of healthcare. As noted previously, the healthcare system in Ghana is structured. At the highest level is the teaching hospitals, followed by regional hospitals, district hospital, polyclinic, health centers or clinic and CHPS. Data were collected from health facilities that are under the auspices of Ministry of Health and Ghana Health Service (Collectively referred to as Government facilities) and Christian Health Association of Ghana (collectively termed CHAG facilities). Private health facilities were excluded from the study since the working conditions of nurses at these facilities differ from the counterparts in Government or CHAG facilities. In this study, majority of the facilities were owned by government ($n$ = 155, 85.6%), whereas 26 (14.4% CHAG facilities.

*Recruiting and Training Field Officers*

A total of 112 field officers were recruited across the 16 regions in Ghana through the regional offices of the GRNMA. A dedicated WhatsApp platform was created for the field officers and the research team to facilitate communication relating to the project. A training workshop was organized and held via the Zoom videoconference platform at the convenience of the field officers

who were working as nurses in their respective health facilities. The major area for the training was the data gathering process, including how to maintain data integrity, avoid data contamination as well as ensure ethically responsible research conduct. This was intended to ensure quality data gathering and transmission via a dedicated electronic portal powered by Google.

*Study Variables*

The study variables, operationalized as the services rendered by the health facilities, were decided by the research team members following a series of meetings and consultations with researchers, policy makers and practitioner. The team also took into consideration local health priorities and the demands of the Sustainable Development Goal (SDG) 3. The research team unanimously agreed on the following study variables; (1) outpatient department services, (2) admissions, (3) deliveries, (4) surgical services, (5) reproductive health services, and (6) antenatal clinic (ANC) services. We defined ANC services as healthcare services delivered to pregnant women. While these may include reproductive health services, we also note instances where reproductive health services are delivered to non-pregnant women. Therefore, in this study, we focus on reproductive health services as services accessible to non-pregnant women. The inclusion of delivery services, for instance, was in accordance with indicator 3.1 of the SGD3 (reducing "global maternal mortality ratio to less than 70 per 100,000 live births") and indicator 3.2 (reducing neonatal mortality to at least as low 12 per 1,000 live births……"). Reproductive health service was also included in view of indicator 3.7 of the SDG3: "universal access to sexual and reproductive health care services, including family planning……". Lastly, the focus on antenatal services reflect indicator 3.1 of the SGD3 which is to reduce "global maternal mortality ratio to less than 70 per 100,000 live births" and indicator 3.2 that is concerned about reducing neonatal

mortality to at least as low 12 per 1,000 live births and under-5 mortality to at least as low as 25 per 1,000 live births."

*Timeframe for Project Data*

Data were collected before, during and after the strike period to allow for comparative analyses and discussions across different data collection points, with reference to the strike period. Because the strike lasted for three consecutive days (21st to 23rd September, 2020; Monday to Wednesday), we collected data for the 3-day period to appreciate the scale of the impact of the strike action. Besides, the number of clients or patients who accessed healthcare services differ from day to day. This made it difficult to restrict the data collection to any of the days the strike action occurred. To obtain a baseline data against which to assess the impact of the strike, we collected data for the 3 consecutive days before the strike action (14th to 16th September 2020; Monday to Wednesday). Lastly, data were collected 3 consecutive days after the strike action was called off (28th to 30th September 2020; Monday to Wednesday) to estimate the use of health services following the suspension of the strike action. The data collection lasted for approximately 2 months, spanning 10th October to 3rd December 2020.

*Data collection Procedure*

Data collection was aided by the tool designed by the research team based on the pre-determined study variables discussed previously. Prior to the data collection, institutional permission was sought from the various health facilities with introductory or permission letter issued by the GRNMA national secretariat to the field officers. As stated previously, data were collected for three consecutive days for each data collection period (e.g., before, during and after strike). The field officers completed the hardcopy of the questionnaire for each facility. Thereafter, they were provided with a dedicated link, powered by Google Form, where they inputted and

transmitted the data electronically to a centralized receiver accessible to the research team. The same questionnaire was used across the health facilities. The field officers were informed to input nil or zero where the data sought for does not exist. For example, because CHPS compounds do not conduct surgeries, data on this service will not be available. The electronic form requires that the field officers provide additional information on the region, district, type of facility (e.g., hospital or health center) and ownership of facilities (e.g., Government or CHAG) where data were collected. Regular updates were provided on the WhatsApp page to keep the field officers informed about the submissions received.

*Ethical Consideration*

This is a retrospective study in which data on the number of people who utilized various healthcare services before, during and after strike action by nurses and midwives were gathered. The study did not involve direct human subject engagement. Rather, data were obtained from institutional archives as an aggregate data. The focus was on how many people visited or utilized healthcare services within the time frame above, without focusing on the background or demographics of users of healthcare services. The data collected were also devoid of identifying information relating to the facilities. This means that neither the facility nor clients/patients will be identified. Thus, data were fully anonymized. The data on number of people accessing healthcare services is notably ~~a~~ public data in Ghana. Given the nature of the study, we did not seek IRB approval. The project was underpinned by other relevant ethical considerations in research, including confidentiality, data safety and data protection. Access to data was restricted to the research team or other individuals supporting the project, mainly data analysts. These individuals signed a statement confirming that they would adhere to the study procedures regarding

confidentiality. The data collected were analyzed as regional aggregate data to further delink the healthcare facilities.

*Data Management*

By the end of the data collection process, a total of 191 submissions were received. However, some of the submissions were duplicates, perhaps because of the technical and internet connectivity issues. It was also observed that, some field officers did not provide the exact or absolute number of service users for the study variable. Instead, they provided inscription such as “over 400”, making it difficult to determine the exact number of service users under reference. The dataset was subsequently cleaned by deleting the data anomalies or deviations, leaving a total of 181 submissions for analyses.

*Data Analysis*

The analysis of data proceeded on two key assumptions; (1) health facilities under the various categorization (e.g., hospital, health centers) in a region will be similar with respect to the average number of service users than those outside the region. That is, hospitals in Ashanti region will be similar in terms of the number of service users than hospitals in Volta region. This assumption is centered heavily on the variations in the population distribution across the regions which in turn influence the number of health service consumers; and (2) health facilities falling under a particular category will be similar in terms of the range of the services provided. For example, it was assumed that CHPS compound across the country will offer virtually the same type of health services. In the same vein, hospitals across the country are more likely to render the same set of health services. Any difference should be subtle or negligible. Based on the foregoing, data was analyzed at the health facilities level, segregated by region. That is, hospital data were analyzed on regional basis as were data from health centers.

To proceed, we computed the average number of service users for each region, taking into consideration the type of health facilities. For example, data from the hospitals in Ashanti region were summed and divided by the total number of hospitals that provided data for the study. This resulted in the average number of health service users from hospitals in Ashanti region. In instances where data were available for only one type of health facility in a region, the same data were used since the mean could not be calculated. Although the mean is sensitive to outliers, it is the most widely used descriptive statistics in research and publication. To address problems relating to outliers, we aggregated and analyzed data along regional framework and by nature of health facilities. Data was prepared using the IBM SPSS Version 23 and analyzed using excel. The analyses involve mostly descriptive statistics. We computed the percentage change in the average number of individuals accessing health services before, during and after strike.

## Results

In this section, we presented the results of the data analyses, commencing with the distribution of healthcare facilities. This was followed by the presentation of findings relating to the utilization of healthcare services. This was structured based on the type of health facilities, starting first with hospitals, followed by polyclinics, and lastly health centers. We examined the extent of and change in use of the various health services described under study variables for the various health facility types. This was done separately for each region.

*Distribution of Health Facilities*

As noted previously, data were collected from a total of 181 health facilities. More than half of the facilities were hospitals ($n$ = 93; 51.4%), 64 were health centers (35.4%), 16 were CHPS compounds (8.8%) and 8 were polyclinics (4.4%). Table 1 showed the regional breakdown of the number of health facilities included in the project. With respect to the 93 hospitals, majority were in Ashanti region ($n$ = 12), followed by Volta and Upper West ($n$ = 9

each) and Upper East and Western (*n* = 8 each), with only in Ahafo region. Of the 64 health centers, majority were in Ashanti region (*n* = 12), followed by Central region (*n* = 13) and Upper West (*n* = 8), with only from Northern region. Of the 16 CHPS compounds, Upper West region contributed to six, followed by Western region (*n* = 4). There was no data from CHPS compounds located in several regions, including Ashanti, Oti, Bono, Eastern and Ahafo. Data were obtained from two polyclinics in Greater Accra and one each in the following regions: Ashanti, Western North, Bono, Upper West, North East and Central.

Table 1: *Number and type of health facilities across the regions*

| **Regions** | **Frequency** | **%** | **Regions** | **Frequency** | **%** |
|---|---|---|---|---|---|
| **Ashanti** | | | **Greater Accra** | | |

| | | | | | |
|---|---|---|---|---|---|
| Hospital | 12 | 48 | Hospital | 6 | 54.5 |
| Polyclinic | 1 | 4 | Polyclinic | 2 | 18.2 |
| Health Center | 12 | 48 | Health Center | 2 | 18.2 |
| CHPS | 0 | 0 | CHPS | 1 | 9.1 |
| **Total** | **25** | **100** | **Total** | **11** | **100** |
| | | | | | |
| **Eastern** | | | **Ahafo** | | |
| Hospital | 5 | 100 | Hospital | 1 | 100 |
| **Total** | **5** | **100** | **Total** | **1** | **100** |
| | | | | | |
| **Western** | | | **Western North** | | |
| Hospital | 8 | 53.3 | Hospital | 4 | 36.4 |
| Polyclinic | 0 | 0 | Polyclinic | 1 | 9.1 |
| Health Center | 3 | 20 | Health Center | 5 | 45.5 |
| CHPS | 4 | 26.7 | CHPS | 1 | 9.1 |
| **Total** | **15** | **100** | **Total** | **11** | **100** |
| | | | | | |
| **Oti** | | | **Bono East** | | |
| Hospital | 4 | 100 | Hospital | 3 | 100 |
| **Total** | **4** | **100** | **Total** | **3** | **100** |
| | | | | | |
| **Volta** | | | **Bono** | | |
| Hospital | 9 | 81.8 | Hospital | 6 | 66.7 |
| Polyclinic | 0 | 0 | Polyclinic | 1 | 11.1 |
| Health Center | 2 | 18.2 | Health Center | 2 | 22.2 |
| **Total** | **11** | **100** | **Total** | **9** | **100** |
| | | | | | |
| **Northern** | | | **Savannah** | | |
| Hospital | 7 | 87.5 | Hospital | 2 | 18.2 |
| Health Center | 1 | 12.5 | Health Center | 6 | 54.5 |
| CHPS | 0 | | CHPS | 3 | 27.3 |
| **Total** | **8** | **100** | **Total** | **11** | **100** |
| | | | | | |
| **Upper West** | | | **Upper East** | | |
| Hospital | 9 | 37.5 | Hospital | 8 | 66.7 |
| Polyclinic | 1 | 4.2 | Polyclinic | 0 | 0 |
| Health Center | 8 | 33.3 | Health Center | 4 | 33.3 |
| CHPS | 6 | 25.0 | CHPS | 0 | 0 |
| **Total** | **24** | **100** | **Total** | **12** | **100** |
| | | | | | |
| **Central** | | | **North East** | | |
| Hospital | 7 | 31.8 | Hospital | 2 | 22.2 |
| Polyclinic | 1 | 4.5 | Polyclinic | 1 | 11.1 |
| Health Center | 13 | 59.1 | Health Center | 6 | 66.7 |
| CHPS Compound | 1 | 4.5 | CHPS | 0 | 0 |
| **Total** | **22** | **100** | **Total** | 9 | 100 |

**Access to Health Services at Hospitals Before, During and After Strike**

*Outpatient Department Attendance*

As shown in Table 2, the average number of patients accessing healthcare services at the outpatient department (OPD) reduced significantly during the strike, compared with before the strike. Taking Ahafo region as an example, an average of 363 patients attended the OPD. This number reduced drastically to 41 during the strike period. The percentage decrease in the mean OPD attendance for the regions ranges from 45.12% (Oti region) to 97.95% (North East region), whereas the percentage increase in mean OPD attendance after the strike was called off ranges from 83.40% (Bono region) to +100% (e.g., Ashanti region).

Table 2: *Percentage change in the mean OPD attendance during and after strike*

| **Region** | **Before Strike** | **During Strike** | | **After Strike** | |
|---|---|---|---|---|---|
| | Mean OPD Attendance | Mean OPD Attendance | % Δ from Before Strike | Mean OPD Attendance | % Δ from During Strike |
| Ahafo | 363 | 41 | -88.70 | 441 | +100 |
| Ashanti | 341.67 | 163.92 | -52.02 | 339.67 | +100 |
| Bono | 551.83 | 268.17 | -51.40 | 491.83 | 83.40 |
| Bono East | 702 | 383 | -45.44 | 712.67 | 86.08 |
| Central | 370.14 | 116.86 | -68.43 | 404.14 | +100 |
| Eastern | 426.8 | 159.6 | -62.61 | 477 | +100 |
| Greater Accra | 425.33 | 81 | -80.96 | 464.17 | +100 |
| North East | 317.5 | 6.5 | -97.95 | 341.5 | +100 |
| Northern | 475 | 26.57 | -94.40 | 660.14 | +100 |
| Oti | 333.5 | 183 | -45.12 | 361.5 | 97.54 |
| Savannah | 261 | 87 | -66.67 | 314.5 | +100 |
| Upper East | 293.38 | 100.63 | -65.70 | 311.13 | +100 |
| Upper West | 157.89 | 16.11 | -89.80 | 171.56 | +100 |
| Volta | 440.78 | 167.67 | -61.96 | 480.22 | +100 |
| Western | 397.37 | 183.13 | -53.91 | 435.32 | +100 |
| Western North | 130.75 | 56.25 | -56.98 | 154 | +100 |

*Admissions*

From the data presented in Table 3, the strike impacted negatively on hospital admissions. The average number of patients on admissions before and after the strike was more than twice the average number during the strike period. The percentage decrease in the mean admissions ranges from 46.42% (Bono region) to 94.58% (Upper West region). All the regions registered an increase in admissions after the strike was called off, with a percentage increase ranging 77.13% (Central region) to +100% (e.g., Greater Accra).

Table 3: *Percentage change in the mean admissions during and after strike*

| **Region** | **Before Strike** | **During Strike** | | **After Strike** | |
|---|---|---|---|---|---|
| | Mean Admission | Mean Admission | % Δ from Before Strike | Mean Admission | % Δ from During Strike |
| Ahafo | 71 | 5 | -92.96 | 73 | +100 |
| Ashanti | 57.5 | 26 | -54.78 | 46.67 | +79.5 |
| Bono | 116.33 | 62.33 | -46.42 | 110 | +76.48 |
| Bono East | 109.67 | 30.67 | -72.03 | 101.67 | +100 |
| Central | 81.71 | 36.86 | -54.89 | 65.29 | +77.13 |
| Eastern | 52.2 | 11.2 | -78.54 | 54.8 | +100 |
| Greater Accra | 50.17 | 11.83 | -76.42 | 52.67 | +100 |
| North East | 167.5 | 4 | -97.61 | 189 | +100 |
| Northern | 86 | 6 | -93.02 | 129.14 | +100 |
| Oti | 88.75 | 35 | -60.56 | 77.25 | +100 |
| Savannah | 42 | 20.5 | -51.19 | 54.5 | +100 |
| Upper East | 89 | 14.88 | -83.28 | 77.88 | +100 |
| Upper West | 75.78 | 4.11 | -94.58 | 67.33 | +100 |
| Volta | 120.33 | 32.11 | -73.31 | 105.33 | +100 |
| Western | 117.5 | 23.63 | -79.89 | 119.38 | +100 |
| Western North | 62.5 | 15.25 | -75.60 | 66 | +100 |

*Deliveries Conducted*

Table 4 showed that during the strike period, the average number of deliveries conducted across the regions was very low compared to the average number of deliveries conducted prior to the strike and after the strike. In Northern region, for example, an average of 25 deliveries were conducted prior to the strike. This reduced to 2 during the strike period but increased to 40 after the strike was called off. In terms of percentage change in deliveries conducted, Savannah region reported the lowest decrease (10%), whereas Bono East registered the highest decrease (93.61%). With the exception of Greater Accra region, all the other regions recorded more than 100% increase in mean deliveries after the strike was called relative to the strike period.

Table 4: *Percentage change in the mean deliveries conducted during and after strike*

| **Region** | **Before Strike** | **During Strike** | | **After Strike** | |
|---|---|---|---|---|---|
| | Mean Delivery | Mean Delivery | % Δ from Before Strike | Mean Delivery | % Δ from During Strike |
| Ahafo | 18 | 3 | -83.33 | 25 | +100 |
| Ashanti | 12.33 | 1.45 | -88.24 | 10.67 | +100 |
| Bono | 17.83 | 6.17 | -65.40 | 16.33 | +100 |
| Bono East | 11 | 0.67 | -93.91 | 12.67 | +100 |
| Central | 12.43 | 3.43 | -72.40 | 10.29 | +100 |
| Eastern | 14.4 | 3.4 | -76.39 | 12.8 | +100 |
| Greater Accra | 22.17 | 8.83 | -60.17 | 16.83 | 90.60 |
| North East | 19.5 | 4.5 | -76.92 | 20 | +100 |
| Northern | 25.71 | 2 | -92.22 | 40 | +100 |
| Oti | 9.5 | 6.25 | -34.21 | 14 | +100 |
| Savannah | 5 | 4.5 | -10 | 12 | +100 |
| Upper East | 38.5 | 5.38 | -86.03 | 17 | +100 |
| Upper West | 16.44 | 5 | -69.59 | 17 | +100 |
| Volta | 12.33 | 3 | -75.67 | 11.22 | +100 |
| Western | 12.13 | 5.63 | -53.59 | 13 | +100 |
| Western North | 7.75 | 1.25 | -83.87 | 9 | +100 |

*Surgeries Performed*

The data presented in Table 5 revealed that, the average number of surgeries performed decreased across the regions during the strike period, ranging from 67.27% (Savannah region) to 100% (e.g., North East). As can be seen in Table 5, no surgeries were performed in Ahafo and North East regions during the strike period. Whereas there was an increase in surgeries performed in most regions when the strike was called off relative to the strike period (≥ 100% percentage increase in mean), only Savannah region recorded a decrease of 55.56%.

Table 5: *Percentage change in the mean surgeries performed during and after strike*

| **Region** | **Before Strike** | **During Strike** | | **After Strike** | |
|---|---|---|---|---|---|
| | Mean Surgeries | Mean Surgeries | % Δ from Before Strike | Mean Surgeries | % Δ from During Strike |
| Ahafo | 1 | 0 | -100 | 2 | 100 |
| Ashanti | 6.25 | 1.75 | -72 | 5.42 | +100 |
| Bono | 18.33 | 4.17 | -77.25 | 19.17 | +100 |
| Bono East | 17.33 | 2.33 | -86.56 | 16 | +100 |
| Central | 7.14 | 1.29 | -81.93 | 6.57 | +100 |
| Eastern | 18 | 1.4 | -92.22 | 23.2 | +100 |
| Greater Accra | 12.67 | 3.33 | -73.71 | 42.5 | +100 |
| North East | 5 | 0 | -100 | 2.5 | 100 |
| Northern | 8.57 | 0.86 | -89.97 | 12.43 | +100 |
| Oti | 5.25 | 1.5 | -71.43 | 4.75 | +100 |
| Savannah | 27.5 | 9 | -67.27 | 4 | -55.56 |
| Upper East | 7.25 | 0.5 | -93.10 | 12 | +100 |
| Upper West | 6.22 | 1.22 | -80.39 | 6.33 | +100 |
| Volta | 8.33 | 0.89 | -89.32 | 8.56 | +100 |
| Western | 10.75 | 2.75 | -74.42 | 10.25 | +100 |
| Western North | 3.75 | 0.75 | -80 | 5.5 | +100 |

*Reproductive Health Services*

Across the regions, it was observed that the average number of people who accessed reproductive health services dwindled dramatically during the strike period, compared with the periods before and after strike (Table 6). In three of the regions (i.e., Ahafo, Central and Northern), no one accessed reproductive health services during the strike period. The decrease in mean reproductive service use during the strike period, relative to before the strike ranges from 45.74% (Bono region) to 99.80% (Volta region). In contrast, the regions experienced a significant increase (i.e., ≥ 60.74 %) in reproductive service use following the suspension of the strike, except in Savannah region where there was 96% decrease.

Table 6: *Percentage change in the mean reproductive service use (RSU) during and after strike*

| **Region** | **Before Strike** | **During Strike** | | **After Strike** | |
|---|---|---|---|---|---|
| | Mean RSU | Mean RSU | % Δ from Before Strike | Mean RSU | % Δ from During Strike |
| Ahafo | 68 | 0 | -100 | 230 | 100 |
| Ashanti | 88.5 | 8.92 | -89.92 | 119.5 | +100 |
| Bono | 43 | 23.33 | -45.74 | 37.5 | 60.74 |
| Bono East | 63.67 | 5 | -92.15 | 48.67 | +100 |
| Central | 66.5 | 0 | -100 | 55.86 | 100 |
| Eastern | 21.6 | 0.4 | -98.15 | 59.8 | +100 |
| Greater Accra | 78.83 | 13.5 | -82.87 | 76.17 | +100 |
| North East | 107 | 0 | -100 | 161.5 | 100 |
| Northern | 38.33 | 0 | -100 | 60.33 | 100 |
| Oti | 27 | 1 | -96.30 | 37.75 | +100 |
| Savannah | 14 | 25 | 78.57 | 1 | -96 |
| Upper East | 51.75 | 6 | -88.41 | 55.38 | +100 |
| Upper West | 33.56 | 1.11 | -96.69 | 31.89 | +100 |
| Volta | 77.78 | 0.11 | -99.86 | 66.56 | +100 |
| Western | 58.25 | 2.25 | -96.14 | 65 | +100 |
| Western North | 59.5 | 16.25 | -72.69 | 53.25 | +100 |

*Antenatal Clinic Services*

The average number of people who accessed antenatal clinic (ANC) services decreased greatly during the period of strike, compared with the period before and after the strike (Table 7). Ashanti region recorded the least percentage decrease in mean antenatal use (76.13%), whereas North East and Northern regions registered the highest decrease (100%). Relatedly, across the regions, there was more than 100% increase in antenatal service use following the suspension of the strike action, relative to during the strike.

*Table 7: Percentage change in the mean antenatal clinic service use (ANC) during and after strike*

| **Region** | **Before Strike** | **During Strike** | | **After Strike** | |
|---|---|---|---|---|---|
| | Mean ASU | Mean ASU | % Δ from Before Strike | Mean ASU | % Δ from During Strike |
| Ahafo | 135 | 9 | -93.33 | 175 | +100 |
| Ashanti | 68.42 | 16.33 | -76.13 | 79.67 | +100 |
| Bono | 89 | 13.17 | -85.20 | 115.17 | +100 |
| Bono East | 215 | 67.67 | -68.53 | 234.33 | +100 |
| Central | 58.43 | 6.57 | -88.76 | 70.43 | +100 |
| Eastern | 63.2 | 2.2 | -96.52 | 124.6 | +100 |
| Greater Accra | 87.83 | 19.67 | -77.60 | 98 | +100 |
| North East | 150 | 0 | -100 | 187 | +100 |
| Northern | 164 | 0 | -100 | 291.71 | +100 |
| Oti | 60 | 16.75 | -72.08 | 70.75 | +100 |
| Savannah | 41.5 | 4.5 | -89.16 | 37.5 | +100 |
| Upper East | 27 | 0.5 | -98.15 | 30.25 | +100 |
| Upper West | 29.89 | 0.78 | -97.39 | 39.22 | +100 |
| Volta | 85.33 | 0.75 | -99.12 | 105.67 | +100 |
| Western | 104.5 | 24.88 | -76.19 | 117.5 | +100 |
| Western North | 34.5 | 1 | -97.10 | 48 | +100 |

## Access to Health Services at Polyclinics during Strike

*Outpatient Department Attendance*

The data on OPD attendance in Table 8 revealed that the average number of people accessing OPD services reduced drastically during the strike period, compared with before strike. The percentage decrease in OPD attendance ranges from 23.44% (Central region) to 100% in Bono region. Taking Ashanti region as example, before the strike, 299 people accessed OPD services. However, this dropped to 55 during the strike period, representing 81.61% decrease. The results further showed that, OPD attendance increased above 100% across the regions when the strike was called off.

Table 8: *Percentage change in outpatient department attendance during and after strike*

| **Region** | **Before Strike** | **During Strike** | | **After Strike** | |
|---|---|---|---|---|---|
| | Mean OPD Attendance | Mean OPD Attendance | % Δ from Before Strike | Mean OPD Attendance | % Δ from During Strike |
| Ashanti | 299 | 55 | -81.61 | 409 | +100 |
| Bono | 102 | 0 | -100 | 110 | +100 |
| Central | 128 | 98 | -23.44 | 133 | +100 |
| Greater Accra* | 66 | 7.5 | -88.64 | 38.5 | +100 |
| North East | 142 | 0 | -100 | 144 | +100 |
| Upper West | 65 | 8 | -87.69 | 62 | +100 |
| Western North | 17 | 24 | 41.18 | 14 | +100 |

**= mean/average data*

*Admissions*

As shown in Table 9, the average number of patients on admissions declined during the strike period across the regions. For example, In Bono, Greater Accra and North East regions, there were zero admissions during the strike period. All the regions witnessed an increase in admissions after the strike, ranging from 41.67% to +100%.

Table 9: *Percentage change in mean admissions during and after strike*

| Region | Before Strike | During Strike | | After Strike | |
|---|---|---|---|---|---|
| | Mean Admissions | Mean Admissions | % Δ from Before Strike | Mean Admissions | % Δ from During Strike |
| Ashanti | 29 | 10 | -65.52 | 42 | +100 |
| Bono | 4 | 0 | -100 | 9 | +100 |
| Central | 20 | 12 | -40 | 7 | 41.67 |
| Greater Accra* | 13 | 0 | -100 | 4 | +100 |
| North East | 18 | 0 | -100 | 2 | +100 |

**= mean/average data*

*Deliveries Conducted*

The average number of deliveries conducted reduced during the strike period, compared with the period before and after the strike (Table 10). In Ashanti region, for instance, 2 deliveries were conducted on average during the strike, whereas the average number of deliveries before and after the strike stood at 5 and 15, respectively. In regions such as Bono and North East, there were no deliveries performed during the strike period. However, all the regions recorded more than 100% increase in deliveries conducted after the strike was called off.

Table 10: *Percentage change in deliveries conducted during and after strike*

| Region | Before Strike | During Strike | | After Strike | |
|---|---|---|---|---|---|
| | Mean Delivery | Mean Delivery | % Δ from Before Strike | Mean Delivery | % Δ from During Strike |
| Ashanti | 5 | 2 | -60 | 15 | +100 |
| Bono | 5 | 0 | -100 | 4 | +100 |
| Central | 4 | 3 | -25 | 8 | +100 |
| Greater Accra* | 1.5 | 0.5 | -66.67 | 0 | +100 |
| North East | 8 | 0 | -100 | 5 | +100 |
| Upper West | 3 | 2 | -33.33 | 4 | +100 |

**= mean/average data*

*Reproductive Health Services*

The result in Table 11 showed that, during the strike, no reproductive services were provided at Ashanti, Bono, Greater Accra and Upper West regions. Only Central region provided reproductive health services during the strike period. In Ashanti and Bono regions, more reproductive services were provided after the strike was called off, compared with before the strike was declared.

Table 11: *Percentage change in reproductive service use (RSU) during and after strike*

| **Region** | **Before Strike** | **During Strike** | | **After Strike** | |
|---|---|---|---|---|---|
| | Mean RSU | Mean RSU | % Δ from Before Strike | Mean RSU | % Δ from During Strike |
| Ashanti | 256 | 0 | -100 | 251 | +100 |
| Bono | 8 | 0 | -100 | 12 | +100 |
| Central | 2 | 5 | +100 | 4 | -20 |
| Greater Accra* | 40 | 0 | -100 | 37 | +100 |
| Upper West | 3 | 0 | -100 | 2 | +100 |

**= mean/average data*

## Access to Health Services at Health Centers During Strike

*Outpatient Department Attendance*

As shown in Table 12, the average number of OPD attendance at health centers across the regions decreased during the strike period. Three of the regions (e.g., Greater Accra, Northern and Western regions) recorded no OPD attendance during the strike. For the remaining regions, the percentage decrease in average OPD attendance ranges from 46.86% (Western North region) to 94.49% (Upper West). Similarly, there was over 100% increase in the average OPD when the strike was called off.

Table 12: *Percentage change in the mean OPD attendance during and after strike*

| **Region** | **Before Strike** | **During Strike** | | **After Strike** | |
|---|---|---|---|---|---|
| | Mean OPD Attendance | Mean OPD Attendance | % Δ from Before Strike | Mean OPD Attendance | % Δ from During Strike |
| Ashanti | 44.25 | 11.08 | -74.96 | 48.92 | +100 |
| Bono | 66 | 12 | -81.82 | 132 | +100 |
| Central | 53.62 | 5.92 | -88.96 | 55.23 | +100 |
| Greater Accra | 36 | 0 | -100 | 31 | +100 |
| North East | 105.33 | 33 | -68.67 | 111.33 | +100 |
| Northern | 16 | 0 | -100 | 12 | +100 |
| Savannah | 10.83 | 3 | -72.30 | 12.17 | +100 |
| Upper East | 40.5 | 2.75 | -93.21 | 37.75 | +100 |
| Upper West | 31.75 | 1.75 | -94.49 | 24.25 | +100 |
| Volta | 82 | 14.5 | -82.32 | 94.5 | +100 |
| Western | 18.67 | 0 | -100 | 26 | +100 |
| Western North | 28.6 | 15.2 | -46.85 | 36.2 | +100 |

*Deliveries Conducted*

Table 13 showed that the average deliveries conducted in the health centers reduced significantly during the strike period. The percentage decrease ranges from 14.29% (Western North) to 100% (e.g., Northern region). Following the suspension of the strike, the average

deliveries conducted increased beyond 100% from during the strike period for all but one region (Northern region) where there was no deliveries conducted.

Table 13: *Percentage change in the mean deliveries conducted during and after strike*

| **Region** | **Before Strike** | **During Strike** | | **After Strike** | |
|---|---|---|---|---|---|
| | Mean Deliveries | Mean Deliveries | % Δ from Before Strike | Mean Deliveries | % Δ from During Strike |
| Ashanti | 2.75 | 0.58 | -78.91 | 2.58 | +100 |
| Bono | 0.5 | 0 | -100 | 1 | 100 |
| Central | 1.69 | 0.67 | -60.36 | 2.69 | +100 |
| Greater Accra | 1 | 0.5 | -50 | 4 | +100 |
| North East | 0.83 | 0.17 | -79.52 | 1 | 100 |
| Northern | 1 | 0 | -100 | 0 | 0 |
| Savannah | 1.17 | 1.5 | 28.21 | 1 | +100 |
| Upper East | 1.75 | 0.75 | -57.14 | 1.5 | +75 |
| Upper West | 0.5 | 0.38 | -24 | 0.75 | +100 |
| Volta | 2 | 0 | -100 | 3.5 | +100 |
| Western | 1.33 | 0 | -100 | 0.33 | +100 |
| Western North | 1.4 | 1.2 | -14.29 | 1.6 | +33 |

## Discusion

Nurses and midwives are extremely important in the provision of healthcare services in Ghana, as stated previously. The health system is functional and operational due to the contributions of nurses and midwives. As a result, the strike action embarked on by the GRNMA from 21st septermber 2020 to 23rd September 2020 had significant impacts on access to health care services.

The results of the study showed that access to healthcare services decreased significantly across health facilities during the strike period. The average number of patients on admission before and after strike were more than twice the average number during the strike period. The reduction in healthcare deliveries was evident across the range of healthcare services investigated, including OPD attendance, admissions, deliveries, and surgeries. The findings reported here largely support previous studies from other African countries, including Kenya (Njuguna, 2015), Nigeria (Oleribe et al., 2018) and Egypt (Shukla, 2012) that showed a reduction in healthcare services utilization following strike actions by nurses and other health professionals.

A major strength of the current study is the wider focus. Unlike the narrowed focus of previous studies (Oleribe et al., 2016; Shukla, 2012; Waithaka et al., 2020), this study focused on the impact of strike on a range of healthcare services from the 16 administrative regions of Ghana. Across the regions, there was a significant reduction in reproductive health service utilization. In three of the regions (i.e., Ahafo, Central and Northern), no one accessed reproductive health services during the strike period. The average number of people who utilized antenatal services decreased during the period of strike, compared with the period before and after the strike. In the North East and Northern regions, for example, there was ~~a~~ zero use of antenatal services during the strike. Our study has shown that strike action by nurses and midwives has the propensity to

negatively impact access to a range of healthcare services provided by different units or departments in different administrative or geographical locations.

The decrease in healthcare access could be attributed to the fact that nurses and midwives are the “basic unit” of the healthcare system such that in their absence, the public is not able to initiate help-seeking behavior. Indeed, nurses activate the healthcare system through the provision of basic health services such as temperature, blood pressure and blood glucose that form the foundation for advanced and detailed healthcare services. Besides, nurses provide major support to other health professionals in rendering healthcare services. Surgeries, for instance, cannot be performed by only surgeons without the professional skills of theatre nurses. Surgical cases require admissions before and after the surgical procedure. Nurses generally manage the admission processes. Without adequate number of nurses to care for patients before and after surgery, undesirable surgical outcomes, including complication and wound infection, may manifest quite easily and quickly. Therefore, the absence of nurses tends to create a significant professional gap that other health professionals, by virtue of their training and specialization, are not able to immediately and appropriately fulfill their roles. Because the public is aware of the duties and responsibilities nurses and midwives play in the health system, news about their strike could affect help-seeking decision from healthcare facilities.

The reduction in access to healthcare services have enormous consequences, as demonstrated by previous studies. For example, strike action by healthcare workers has been linked to increased mortalities (Kaguthi et al., 2020). The absence or limited supply of vaccination services occasioned by strike action (Njuguna, 2015) could complicate efforts to improve the health of children and adolescent as well as preventing death from childhood diseases. Although data were not obtained on service users; however, previous studies have shown that social and

economically disadvantaged groups are extremely vulnerable to the negative repercussions of strike as they are unable to pay for services in a private hospital (Irimu et al., 2018). These individuals may resort to the use of herbal preparations and self-medication that often exacerbate their conditions, culminating into increased morbidity and mortality (Irimu et al., 2018; Oleribe et al., 2018; Waithaka et al., 2020).

A major caveat in the literature is the limited data on access to healthcare services following the suspension of strike action by healthcare professionals. To fill this void, the current study compared healthcare services utilization during and after the strike action by nurses and midwives. As reported previously, service used increased drastically following the cessation of the strike action. The increase in access to healthcare services is a testament of the unmet health needs of the public. The decrease in the provision of healthcare services during the strike period could drive the public towards private healthcare system. However, most Ghanaians cannot afford the services of private healthcare facilities owing to the relatively high cost of treatment(Burger et al., 2012). These individuals are left with the option of waiting on the news of resumption of work by nurses and midwives so that they can access healthcare. The drastic increase in the use of healthcare services could be due to several factors, including the fear of another strike. Indeed, the strike was called off following an injunction order secured from a court of competent jurisdiction by the National Labor Commission (NLC) of Ghana, who questioned the legitimacy of the strike. The NLC, which is mandated to address labor agitation and unrest issues in Ghana, does not negotiate with striking labor unions. Like in other jurisdiction, the labor laws favor strike. However, the processes to do so have always been so onerous and time-consuming that unions in Ghana rarely engage in legally recognized strikes (Baah & Reilly, 2009). The public could be worried that the negotiation between the NLC and GRNMA may fail, thereby providing the motivation for another

strike action. Indeed, instances of failed negotiation between the NLC and labor unions are very common development in Ghana. The public, therefore, saw the immediate resumption of duty as the best opportunity to obtain healthcare services before another strike is announced. There is also the possibility that people visited the health facilities to learn for themselves whether the healthcare delivery process has normalized, following its truncation by the strike actions. In the process, they might have accessed healthcare services, adding to the number of service users after the strike period.

**Limitations of the Study**

The study findings should be evaluated considering the following limitations. The study was designed to gather data from the health facilities located across the 16 administrative regions of Ghana. However, as a typical nature of research, we could not obtain data from all the facilities across the regions or districts. Some heads of facilities did not grant approval for the data gathering process. In some instances, we could not identify individuals interested in supporting the research as field officers. The data reported here were based on facilities that were somewhat conveniently selected. The lack of random selection of the health facilities limits the generalization of the findings reported in this study. Given the nature of the research design, particularly the data collection method and data collected, it was impossible to determine whether and to what extent other factors beyond the strike action contributed to the decrease in access to healthcare during strike period and an increase post-strike.

**Conclusions**

The strike action by the nurses and midwives in Ghana significantly reduced access to a range of health services, including reproductive health services, deliveries, and antenatal services.

The distribution of the impacts of the strike action across the regions is largely uniform. Likewise, the impact of the strike action extended to various types of health facilities, hospitals, polyclinics and health centers. The far-reaching impact suggest clearly that strike action by nurses and midwives will derail the efforts and investment made towards achieving the universal healthcare coverage by the Government of Ghana and development partners. It is also imperative to state that the attainment of the goal 3 (health and well-being) of SDG in Ghana will be negatively affected with strike action by nurses and midwives. This understanding calls for proactive measures by the Government of Ghana and relevant organizations to respond adequately and appropriately to the needs and concerns of nurses and midwives as the largest healthcare workforce. Doing so requires some practical steps such as periodic assessment of the wellbeing and conditions of service of nurses and midwives and provide the necessary support system that accommodate existing socioeconomic conditions. Increasing cost of goods and services, for example, should be accompanied by a corresponding increase in salary or financial incentives. This basic economic principle would mitigate against the financial challenges suffered by nurses and midwives, significantly reducing agitation for improved conditions of services amidst economic hardship induced by the raising inflation and cost of petroleum products.

References

Agyepong, I. A., Kodua, A., Adjei, S., & Adam, T. (2012). When 'solutions of yesterday become problems of today': crisis-ridden decision making in a complex adaptive system (CAS)—the Additional Duty Hours Allowance in Ghana. *Health Policy and Planning*, *27*(suppl_4), iv20-iv31.

Aro, S., & Hosia, P. (1987). Effects of a doctors' strike on primary care utilization in Varkaus, Finland. *Scandinavian Journal of Primary Health Care*, *5*(4), 245-251.

Baah, A. Y., & Reilly, B. (2009). An empirical analysis of strike durations in Ghana from 1980 to 2004. *Labour*, *23*(3), 459-479.

Bhuiyan, M., & Machowski, A. (2012). Impact of 20-day strike in Polokwane hospital (18 August-6 September 2010). *South African Medical Journal*, *102*(9), 755-756.

Burger, N. E., Kopf, D., Spreng, C. P., Yoong, J., & Sood, N. (2012). Healthy firms: constraints to growth among private health sector facilities in Ghana and Kenya. *PloS one*, *7*(2), e27885.

Furnivall, D., Bottle, A., & Aylin, P. (2018). Retrospective analysis of the national impact of industrial action by English junior doctors in 2016. *BMJ Open*, *8*(1), e019319.

Ghana Health Service[GHS]. (2003). *The Status of Referrals in Three Districts in Ghana: Analysis of Referral Pathways for Children under Five. The Basic Support for Institutionalizing Child Survival Project (BASICS II) for the United States Agency for International Development, and Ghana Health Service*.

Ghana Health Service [GHS]. (2018). *articles The Health Sector in Ghana. Facts and Figures 2018om*. https://open.africa/dataset/4176f749-cfa8-4e32-9418-86cef78f9db6/resource/0bcf9b54-3e35-4543-95cd-fd4de953edff/download/factsfigures_2018.pdf

Gyamfi, G. D. (2011). Assessing the effects of industrial unrest on Ghana health service: A case study of nurses at Korle-Bu teaching hospital. *International Journal of Nursing and Midwifery*, *3*(1), 1-5.

Irimu, G., Ogero, M., Mbevi, G., Kariuki, C., Gathara, D., Akech, S., Barasa, E., Tsofa, B., & English, M. (2018). Tackling health professionals' strikes: an essential part of health system strengthening in Kenya. *BMJ Global Health*, *3*(6).

Isangula, K. (2012). Activism during doctor's strike in Tanzania, success and setbacks. SSRN Electronic Journal *Sikika Journal*. Available a http://dx.doi.org/10.2139/ssrn.2129021

Kaguthi, G. K., Nduba, V., & Adam, M. B. (2020). The impact of the nurses', doctors' and clinical officer strikes on mortality in four health facilities in Kenya. *BMC Health Services Research*, *20*(1), 1-10.

Manthous, C. A. (2014). Labor unions in medicine: The intersection of patient advocacy and self-advocacy. *Medical care*, *52*(5), 387-392.

Njuguna, J. (2015). Impact of health workers' strike in August 2014 on health services in Mombasa County Referral Hospital, Kenya. *Journal of Health Care for the Poor And Underserved*, *26*(4), 1200-1206.

Oleribe, O. O., Ezieme, I. P., Oladipo, O., Akinola, E. P., Udofia, D., & Taylor-Robinson, S. D. (2016). Industrial action by healthcare workers in Nigeria in 2013–2015: an inquiry into causes, consequences and control—a cross-sectional descriptive study. *Human resources for Health*, *14*(1), 1-10.

Oleribe, O. O., Udofia, D., Oladipo, O., Ishola, T. A., & Taylor-Robinson, S. D. (2018). Healthcare workers' industrial action in Nigeria: a cross-sectional survey of Nigerian physicians. *Human Resources for Health*, *16*(1), 1-10.

Ruiz, M., Bottle, A., & Aylin, P. (2013). A retrospective study of the impact of the doctors' strike in England on 21 June 2012. *Journal of the Royal Society of Medicine*, *106*(9), 362-369.

Scanlon, M. L., Maldonado, L. Y., Ikemeri, J. E., Jumah, A., Anusu, G., Chelagat, S., Keter, J. C., Songok, J., Ruhl, L. J., & Christoffersen-Deb, A. (2021). 'It was hell in the community': a qualitative study of maternal and child health care during health care worker strikes in Kenya. *International Journal for Equity in Health*, *20*(1), 1-12.

Sheahan, K. H., Kennedy, A. G., Tompkins, B. J., Repp, A. B., & Gilbert, M. P. (2021). The Impact of a Nursing Strike on Glycemic Control in Hospitalized Patients with Diabetes. *Cureus*, *13*(6).

Shukla, S. (2012). Violence against doctors in Egypt leads to strike action. *The Lancet*, *380*(9852), 1460.

Van Rensburg, A. J., & van Rensburg, D. J. (2013). Nurses, industrial action and ethics: considerations from the 2010 South African public-sector strike. *Nursing Ethics*, *20*(7), 819-837.

Waithaka, D., Kagwanja, N., Nzinga, J., Tsofa, B., Leli, H., Mataza, C., Nyaguara, A., Bejon, P., Gilson, L., & Barasa, E. (2020). Prolonged health worker strikes in Kenya-perspectives and experiences of frontline health managers and local communities in Kilifi County. *International Journal for Equity in Health*, *19*(1), 23.

Supplementary Files

File 1: Raw data used for the analysis.

Click here to access/download
**Supporting Information**
File_ Raw data used for analysis..xls

# A Multi-Level Impact Analysis of Strike Action by Ghana Registered Nurses and Midwives on Access to and Utilization of Healthcare Services

Perpetual Ofori Ampofo[1]; David Tenkorang-Twum[1,2]; Samuel Adjorlolo[3,4]; Margaretta Gloria Chandi[5]; Francis KwakuWuni[6], Ernestina Asiedu[7], Vida Ami Kukula[8], Sampson Opoku[9]

1. Ghana Registered Nurses and Midwives' Association of Ghana. Head Office, Okponglo, Accra, Ghana.

2. Department of Adult Health, School of Nursing and Midwifery, University of Ghana, Legon, Accra, Ghana.

3. Department of Mental Health, School of Nursing and Midwifery, University of Ghana, Legon, Accra, Ghana.

4. Research and Grant Institute of Ghana, P. Box LG 1004, Legon Post Office, Accra, Ghana.

5. GA West Municipal health directorate, P. O .Box AM187, Accra, Ghana.

6. Regional hospital, P.O. Box 26, Bolgatanga.

7. Department of Child and Maternal Health, School of Nursing and Midwifery, University of Ghana, Accra, Ghana.

8. Dodowa Health Research Center, Dodowa, Accra, Ghana.

9. P. O. Box KF 735. Koforidua, Eastern Region, Ghana.

**Corresponding Author**

Dr. Samuel Adjorlolo, Department of Mental Health, School of Nursing and Midwifery, College of Health Sciences, University of Ghana, P. O. Box LG 43, Legon, Accra, Ghana; email: sadjorlolo@ug.edu.gh

**A Multi-Level Impact Analysis of Strike Action by Ghana Registered Nurses and Midwives on Access to and Utilization of Healthcare Services**


## Abstract

**Background**: As the largest professional group, nurses and midwives play instrumental roles in healthcare delivery, supporting the smooth administration and operation of the health system. Consequently, the withdrawal of nursing and midwifery services via strike action has direct and indirect detrimental effects on access to healthcare.

**Objective**: The current study examined the impact of strike action by nurses and midwives with respect to access to and utilization of health services.

**Method:** Data were collected retrospectively from a total of 181 health facilities from all the 16 administrative regions of Ghana, with the support of field officers. Because the strike lasted for 3 days, the data collection span three consecutive days before the strike, three days of the strike and three consecutive days after the strike. Data analysis was focused comparing the utilization of healthcare services before, during and after strike. Data were analysed and presented on the various healthcare services. This was done separately for the health facility type and the 16 administrative regions.

**Findings**: The results showed that; (1) the average number of patients or clients who accessed healthcare services reduced drastically during the strike period, compared with before the strike. Majority of the regions recorded more than 70% decrease in service use during the strike period; (2) the average number of patients or clients who accessed healthcare services after the strike increased by more than 100% across majority of the regions.

**Conclusion**: The study showed that strike action by nurses and midwives negatively affected access to and utilization of healthcare services.

**Keywords**: Strike, nurses and midwives, healthcare access and use, Ghana

## Introduction

The services provided by nurses and midwives are essential in the attainment of universal healthcare coverage; hence the withdrawal of such services via strike action has direct and indirect detrimental effects (Van Rensburg & van Rensburg, 2013). The current study examines the impact of strike action declared and undertaken by members of Ghana Registered Nurses and Midwives Association (GRNMA), the umbrella body of nurses and midwives in Ghana. Strike is the last weapon used by employees to fight for salary and improved working conditions. Employees who feel their rights and wellbeing have been violated, interfered with, or disregarded by their employer may decide to strike as the only practical way to get their complaints acknowledged and addressed (Osakede, 2014). Strike actions by healthcare workers take varying forms such as suspension of general healthcare delivery services, provision of only emergency services, cessation of work for few hours, days, weeks and months (Agyepong et al., 2012; Manthous, 2014).

The decision to embark on strike is influenced by a myriad of factors, predominant among them is conditions of service. Illustrating, among Ghanaian nurses, dissatisfaction with working conditions, particularly salary, was cited as the motivating factor for their strike actions. More specifically, the nurses expressed concern about the large wage disparity in reference to the medical doctors, rather than equal pay (Gyamfi, 2011). The connection between remuneration of health workers and the emergence of strike actions is further supported by a study in Nigeria. According to Oleribe et al., (2016), the majority of strikes in Nigeria are primarily motivated by a desire for increased pay. In Tanzania, the request for improved working conditions in the areas of

infrastructure, medications, equipment, and other medical supplies, as well as underpayment of salary and allowances were the reasons for doctors' strike in 2012 (Isangula, 2012). Strike actions by healthcare workers in Egypt in 2011 was intended to persuade authorities to reinforce safety in healthcare facilities, increase healthcare budget, and improve on the deteriorating state of facilities in the country's health care system(Shukla, 2012).

Other factors associated with strike actions include: poor working environment, demotivation among staff, delayed or unfairness in promotions (Gyamfi, 2011; Oleribe et al., 2016; Waithaka et al., 2020). Dissatisfaction with leadership and management has also been implicated in strike action by healthcare workers in Nigeria (Oleribe et al., 2016). Notable among the managerial inefficiencies include delayed promotions and limited educational and training support (Oleribe et al., 2018). Other studies have registered solidarity with a colleague for an unlawful assault, or dismissal as reasons why health workers may embark on strike action (Oleribe et al., 2018; Waithaka et al., 2020).

Restricted access to healthcare, occasioned by strike, is a recipe for increased mortality and morbidity, decreased revenue mobilization and other undesirable outcomes. In Nigeria, strike action by healthcare workers potentiated increased referrals to private health facilities (66.0%) and its resultant challenges such as substandard treatment and high cost of services (Oleribe et al., 2016). In a related study in Kenya involving nurses, Njuguna (2015) reported that the strike action had a negative impact on vaccination services for children. For example, there was a 56.9% decrease in the number of newborns who received vaccinations during the strike in public health facilities. However, the proportion of immunizations in Faith –based hospitals increased by 252%. Scanlon et al. (2021) also established a robust link between strike action by healthcare workers and poor maternity and health care utilization, including ANC and delivery at a health facility

among pregnant women, and delays in a child's first oral polio vaccination in Kenya. In a related Kenyan study, outpatient attendance declined by 64.4%, special clinics attendance by 74.2%, deliveries by 53.5% and inpatient admissions by 57.8% when nurses and other healthcare professionals embarked on strike (Njuguna, 2015). A decrease in health services utilization and admissions during the period of strike by nurses and medical doctors in Kenya contributed to a decrease in mortality rates registered across hospitals in Kenya (Kaguthi et al., 2020; Njuguna, 2015). Similar findings have been reported in high income countries, including US (Sheahan et al., 2021), England (Furnivall et al., 2018; Ruiz et al., 2013) and Finland (Aro & Hosia, 1987). For example, Ruiz et al. (2013) in their study in England found that compared with the non-strike period, emergency admissions fell by 2.4% while the elective admissions decreased by 12.8%. The authors reported a 7.8% drop in the number of outpatients seen by medical staff on the day of the strike and a 45.5% increase in the number of cancelled appointments by NHS hospitals, while accident and emergency attendances dropped by 4.7% (Ruiz et al., 2013).

The GRNMA, like other professional associations and labor unions, has embarked on several strike actions since 1978 as a broader strategy to obtain improved and better conditions of services for her members (Gyamfi, 2011). The most recent strike action embarked on by the GRNMA and allied health professionals recently was from September 21$^{st}$ to 23$^{rd}$, 2020 after negotiations with the Government (employer) for improved conditions of service yielded no results. As noted above, strike actions generally leads to a reduction in the number of people accessing healthcare services (Bhuiyan & Machowski, 2012; Oleribe et al., 2018). However, our understanding of the extent of disruption in access to a range of healthcare services is limited as majority of previous studies have tended to focus their attention on services rendered by a unit or department in hospital or health facility (exceptions see Njuguna, 2015). Ghana's healthcare

delivery operates on three levels, thus primary, secondary, and tertiary. At the primary level, both preventive and promotive services are provided by Community health planning services (CHPS) centers, health centers, and polyclinics. They serve a population of approximately 20,000 people. At the secondary level, curative services are provided by district hospitals and serve an average population of 100,000 to 200,000 people whiles at the tertiary level, curative and rehabilitative services are delivered at the regional/teaching hospitals which serve approximately an average population of about 1.2 million (Ghana Health Service[GHS], 2003). The healthcare delivery system has witnessed an increase in the number of healthcare professionals. For example, nurse-patient ratio has improved from 1:2,172 in 2013 to 1:505 in 2017 with the total number of nursing workforce increasing from 12,245 in 2013 to 58,608 in 2017. Again, the midwife to women in fertile age (WIFA) ratio has improved from 1:1533 to 1:704 with the total number of midwives increasing from 4,185 to 9884 (GHS, 2018). As the largest workforce in the healthcare system, nurses and midwives have significant roles to contribute to the attainment of universal health coverage (UHC). Therefore, understanding how strike actions by these professional groups impact the range of healthcare services available to the public will equip stakeholders to take appropriate steps to respond appropriately and timely to strike notices, avert strike actions to safeguard the health of the population. Second, although nurses and midwives and other health professionals in Ghana do embark on strike, there is no comprehensive and systematically generated evidence on the impact of their strike actions on access to healthcare. The lack of empirical data in this regard makes it extremely difficult for the nurses and midwives association (i.e., GRNMA) to quantify the contributions of her members to healthcare delivery and to make a compelling case for better conditions of service. At the same time, the employer is unable to readily envisage the devastation to access to healthcare delivery occasioned by the strike action of nurses and midwives and other

health professionals. The current study fills this void by investigating the impact of the 2020 strike action by GRNMA and its Allied health professionals on access to a range of healthcare services in Ghana.

*Study Objectives*

The objectives of the study are as follows;

1. Determine the number of people accessing and utilization health services before, during and after strike action by nurses and midwives.
2. Compare the number of people who accessed essential healthcare services before, during and after the strike was called off.

## Method

*Project Setting*

Data were gathered from health facilities located across the 16 regions in Ghana and across all the levels of healthcare. As noted previously, the healthcare system in Ghana is structured. At the highest level is the teaching hospitals, followed by regional hospitals, district hospital, polyclinic, health centers or clinic and CHPS. Data were collected from health facilities that are under the auspices of Ministry of Health and Ghana Health Service (Collectively referred to as Government facilities) and Christian Health Association of Ghana (collectively termed CHAG facilities). Private health facilities were excluded from the study since the working conditions of nurses at these facilities differ from the counterparts in Government or CHAG facilities. In this study, majority of the facilities were owned by government ($n$ = 155, 85.6%), whereas 26 (14.4% CHAG facilities.

*Recruiting and Training Field Officers*

A total of 112 field officers were recruited across the 16 regions in Ghana through the regional offices of the GRNMA. A dedicated WhatsApp platform was created for the field officers and the research team to facilitate communication relating to the project. A training workshop was organized and held via the Zoom videoconference platform at the convenience of the field officers who were working as nurses in their respective health facilities. The major area for the training was the data gathering process, including how to maintain data integrity, avoid data contamination as well as ensure ethically responsible research conduct. This was intended to ensure quality data gathering and transmission via a dedicated electronic portal powered by Google.

*Study Variables*

The study variables, operationalized as the services rendered by the health facilities, were decided by the research team members following a series of meetings and consultations with researchers, policy makers and practitioner. The team also took into consideration local health priorities and the demands of the Sustainable Development Goal (SDG) 3. The research team unanimously agreed on the following study variables; (1) outpatient department services, (2) admissions, (3) deliveries, (4) surgical services, (5) reproductive health services, and (6) antenatal clinic (ANC) services. We defined ANC services as healthcare services delivered to pregnant women. While these may include reproductive health services, we also note instances where reproductive health services are delivered to non-pregnant women. Therefore, in this study, we focus on reproductive health services as services accessible to non-pregnant women. The inclusion of delivery services, for instance, was in accordance with indicator 3.1 of the SGD3 (reducing "global maternal mortality ratio to less than 70 per 100,000 live births") and indicator 3.2 (reducing neonatal mortality to at least as low 12 per 1,000 live births……"). Reproductive health service was also included in view of indicator 3.7 of the SDG3: "universal access to sexual and

reproductive health care services, including family planning……”. Lastly, the focus on antenatal services reflect indicator 3.1 of the SGD3 which is to reduce “global maternal mortality ratio to less than 70 per 100,000 live births” and indicator 3.2 that is concerned about reducing neonatal mortality to at least as low 12 per 1,000 live births and under-5 mortality to at least as low as 25 per 1,000 live births.”

*Timeframe for Project Data*

Data were collected before, during and after the strike period to allow for comparative analyses and discussions across different data collection points, with reference to the strike period. Because the strike lasted for three consecutive days (21st to 23rd September, 2020; Monday to Wednesday), we collected data for the 3-day period to appreciate the scale of the impact of the strike action. Besides, the number of clients or patients who accessed healthcare services differ from day to day. This made it difficult to restrict the data collection to any of the days the strike action occurred. To obtain a baseline data against which to assess the impact of the strike, we collected data for the 3 consecutive days before the strike action (14th to 16th September 2020; Monday to Wednesday). Lastly, data were collected 3 consecutive days after the strike action was called off (28th to 30th September 2020; Monday to Wednesday) to estimate the use of health services following the suspension of the strike action. The data collection lasted for approximately 2 months, spanning 10th October to 3rd December 2020.

*Data collection Procedure*

Data collection was aided by the tool designed by the research team based on the pre-determined study variables discussed previously. Prior to the data collection, institutional permission was sought from the various health facilities with introductory or permission letter issued by the GRNMA national secretariat to the field officers. As stated previously, data were

collected for three consecutive days for each data collection period (e.g., before, during and after strike). The field officers completed the hardcopy of the questionnaire for each facility. Thereafter, they were provided with a dedicated link, powered by Google Form, where they inputted and transmitted the data electronically to a centralized receiver accessible to the research team. The same questionnaire was used across the health facilities. The field officers were informed to input nil or zero where the data sought for does not exist. For example, because CHPS compounds do not conduct surgeries, data on this service will not be available. The electronic form requires that the field officers provide additional information on the region, district, type of facility (e.g., hospital or health center) and ownership of facilities (e.g., Government or CHAG) where data were collected. Regular updates were provided on the WhatsApp page to keep the field officers informed about the submissions received.

*Ethical Consideration*

This is a retrospective study in which data on the number of people who utilized various healthcare services before, during and after strike action by nurses and midwives were gathered. The study did not involve direct human subject engagement. Rather, data were obtained from institutional archives as an aggregate data. The focus was on how many people visited or utilized healthcare services within the time frame above, without focusing on the background or demographics of users of healthcare services. The data collected were also devoid of identifying information relating to the facilities. This means that neither the facility nor clients/patients will be identified. Thus, data were fully anonymized. The data on number of people accessing healthcare services is notably a public data in Ghana. Given the nature of the study, we did not seek IRB approval. The project was underpinned by other relevant ethical considerations in research, including confidentiality, data safety and data protection. Access to data was restricted

to the research team or other individuals supporting the project, mainly data analysts. These individuals signed a statement confirming that they would adhere to the study procedures regarding confidentiality. The data collected were analyzed as regional aggregate data to further delink the healthcare facilities.

*Data Management*

By the end of the data collection process, a total of 191 submissions were received. However, some of the submissions were duplicates, perhaps because of the technical and internet connectivity issues. It was also observed that, some field officers did not provide the exact or absolute number of service users for the study variable. Instead, they provided inscription such as "over 400", making it difficult to determine the exact number of service users under reference. The dataset was subsequently cleaned by deleting the data anomalies or deviations, leaving a total of 181 submissions for analyses.

*Data Analysis*

The analysis of data proceeded on two key assumptions; (1) health facilities under the various categorization (e.g., hospital, health centers) in a region will be similar with respect to the average number of service users than those outside the region. That is, hospitals in Ashanti region will be similar in terms of the number of service users than hospitals in Volta region. This assumption is centered heavily on the variations in the population distribution across the regions which in turn influence the number of health service consumers; and (2) health facilities falling under a particular category will be similar in terms of the range of the services provided. For example, it was assumed that CHPS compound across the country will offer virtually the same type of health services. In the same vein, hospitals across the country are more likely to render the same set of health services. Any difference should be subtle or negligible. Based on the foregoing,

data was analyzed at the health facilities level, segregated by region. That is, hospital data were analyzed on regional basis as were data from health centers.

To proceed, we computed the average number of service users for each region, taking into consideration the type of health facilities. For example, data from the hospitals in Ashanti region were summed and divided by the total number of hospitals that provided data for the study. This resulted in the average number of health service users from hospitals in Ashanti region. In instances where data were available for only one type of health facility in a region, the same data were used since the mean could not be calculated. Although the mean is sensitive to outliers, it is the most widely used descriptive statistics in research and publication. To address problems relating to outliers, we aggregated and analyzed data along regional framework and by nature of health facilities. Data was prepared using the IBM SPSS Version 23 and analyzed using excel. The analyses involve mostly descriptive statistics. We computed the percentage change in the average number of individuals accessing health services before, during and after strike.

## Results

In this section, we presented the results of the data analyses, commencing with the distribution of healthcare facilities. This was followed by the presentation of findings relating to the utilization of healthcare services. This was structured based on the type of health facilities, starting first with hospitals, followed by polyclinics, and lastly health centers. We examined the extent of and change in use of the various health services described under study variables for the various health facility types. This was done separately for each region.

*Distribution of Health Facilities*

As noted previously, data were collected from a total of 181 health facilities. More than half of the facilities were hospitals (*n* = 93; 51.4%), 64 were health centers (35.4%), 16 were CHPS compounds (8.8%) and 8 were polyclinics (4.4%). Table 1 showed the regional

breakdown of the number of health facilities included in the project. With respect to the 93 hospitals, majority were in Ashanti region (*n* = 12), followed by Volta and Upper West (*n* = 9 each) and Upper East and Western (*n* = 8 each), with only in Ahafo region. Of the 64 health centers, majority were in Ashanti region (*n* = 12), followed by Central region (*n* = 13) and Upper West (*n* = 8), with only from Northern region. Of the 16 CHPS compounds, Upper West region contributed to six, followed by Western region (*n* = 4). There was no data from CHPS compounds located in several regions, including Ashanti, Oti, Bono, Eastern and Ahafo. Data were obtained from two polyclinics in Greater Accra and one each in the following regions: Ashanti, Western North, Bono, Upper West, North East and Central.

Table 1: *Number and type of health facilities across the regions*

| Regions | Frequency | % | Regions | Frequency | % |
|---|---|---|---|---|---|
| **Ashanti** | | | **Greater Accra** | | |
| Hospital | 12 | 48 | Hospital | 6 | 54.5 |
| Polyclinic | 1 | 4 | Polyclinic | 2 | 18.2 |
| Health Center | 12 | 48 | Health Center | 2 | 18.2 |
| CHPS | 0 | 0 | CHPS | 1 | 9.1 |
| **Total** | **25** | **100** | **Total** | **11** | **100** |
| | | | | | |
| **Eastern** | | | **Ahafo** | | |
| Hospital | 5 | 100 | Hospital | 1 | 100 |
| **Total** | **5** | **100** | **Total** | **1** | **100** |
| | | | | | |
| **Western** | | | **Western North** | | |
| Hospital | 8 | 53.3 | Hospital | 4 | 36.4 |
| Polyclinic | 0 | 0 | Polyclinic | 1 | 9.1 |
| Health Center | 3 | 20 | Health Center | 5 | 45.5 |
| CHPS | 4 | 26.7 | CHPS | 1 | 9.1 |
| **Total** | **15** | **100** | **Total** | **11** | **100** |
| | | | | | |
| **Oti** | | | **Bono East** | | |
| Hospital | 4 | 100 | Hospital | 3 | 100 |
| **Total** | **4** | **100** | **Total** | **3** | **100** |
| | | | | | |
| **Volta** | | | **Bono** | | |
| Hospital | 9 | 81.8 | Hospital | 6 | 66.7 |
| Polyclinic | 0 | 0 | Polyclinic | 1 | 11.1 |
| Health Center | 2 | 18.2 | Health Center | 2 | 22.2 |
| **Total** | **11** | **100** | **Total** | **9** | **100** |
| | | | | | |
| **Northern** | | | **Savannah** | | |
| Hospital | 7 | 87.5 | Hospital | 2 | 18.2 |
| Health Center | 1 | 12.5 | Health Center | 6 | 54.5 |
| CHPS | 0 | | CHPS | 3 | 27.3 |
| **Total** | **8** | **100** | **Total** | **11** | **100** |
| | | | | | |
| **Upper West** | | | **Upper East** | | |
| Hospital | 9 | 37.5 | Hospital | 8 | 66.7 |
| Polyclinic | 1 | 4.2 | Polyclinic | 0 | 0 |
| Health Center | 8 | 33.3 | Health Center | 4 | 33.3 |
| CHPS | 6 | 25.0 | CHPS | 0 | 0 |
| **Total** | **24** | **100** | **Total** | **12** | **100** |
| | | | | | |
| **Central** | | | **North East** | | |
| Hospital | 7 | 31.8 | Hospital | 2 | 22.2 |
| Polyclinic | 1 | 4.5 | Polyclinic | 1 | 11.1 |
| Health Center | 13 | 59.1 | Health Center | 6 | 66.7 |
| CHPS | 1 | 4.5 | CHPS | 0 | 0 |
| Compound | | | | | |
| **Total** | **22** | **100** | **Total** | 9 | 100 |

**Access to Health Services at Hospitals Before, During and After Strike**

*Outpatient Department Attendance*

As shown in Table 2, the average number of patients accessing healthcare services at the outpatient department (OPD) reduced significantly during the strike, compared with before the strike. Taking Ahafo region as an example, an average of 363 patients attended the OPD. This number reduced drastically to 41 during the strike period. The percentage decrease in the mean OPD attendance for the regions ranges from 45.12% (Oti region) to 97.95% (North East region), whereas the percentage increase in mean OPD attendance after the strike was called off ranges from 83.40% (Bono region) to +100% (e.g., Ashanti region).

Table 2: *Percentage change in the mean OPD attendance during and after strike*

| **Region** | **Before Strike** | **During Strike** | | **After Strike** | |
|---|---|---|---|---|---|
| | Mean OPD Attendance | Mean OPD Attendance | % Δ from Before Strike | Mean OPD Attendance | % Δ from During Strike |
| Ahafo | 363 | 41 | -88.70 | 441 | +100 |
| Ashanti | 341.67 | 163.92 | -52.02 | 339.67 | +100 |
| Bono | 551.83 | 268.17 | -51.40 | 491.83 | 83.40 |
| Bono East | 702 | 383 | -45.44 | 712.67 | 86.08 |
| Central | 370.14 | 116.86 | -68.43 | 404.14 | +100 |
| Eastern | 426.8 | 159.6 | -62.61 | 477 | +100 |
| Greater Accra | 425.33 | 81 | -80.96 | 464.17 | +100 |
| North East | 317.5 | 6.5 | -97.95 | 341.5 | +100 |
| Northern | 475 | 26.57 | -94.40 | 660.14 | +100 |
| Oti | 333.5 | 183 | -45.12 | 361.5 | 97.54 |
| Savannah | 261 | 87 | -66.67 | 314.5 | +100 |
| Upper East | 293.38 | 100.63 | -65.70 | 311.13 | +100 |
| Upper West | 157.89 | 16.11 | -89.80 | 171.56 | +100 |
| Volta | 440.78 | 167.67 | -61.96 | 480.22 | +100 |
| Western | 397.37 | 183.13 | -53.91 | 435.32 | +100 |
| Western North | 130.75 | 56.25 | -56.98 | 154 | +100 |

*Admissions*

From the data presented in Table 3, the strike impacted negatively on hospital admissions. The average number of patients on admissions before and after the strike was more than twice the average number during the strike period. The percentage decrease in the mean admissions ranges from 46.42% (Bono region) to 94.58% (Upper West region). All the regions registered an increase in admissions after the strike was called off, with a percentage increase ranging 77.13% (Central region) to +100% (e.g., Greater Accra).

Table 3: *Percentage change in the mean admissions during and after strike*

| **Region** | **Before Strike** | **During Strike** | | **After Strike** | |
|---|---|---|---|---|---|
| | Mean Admission | Mean Admission | % Δ from Before Strike | Mean Admission | % Δ from During Strike |
| Ahafo | 71 | 5 | -92.96 | 73 | +100 |
| Ashanti | 57.5 | 26 | -54.78 | 46.67 | +79.5 |
| Bono | 116.33 | 62.33 | -46.42 | 110 | +76.48 |
| Bono East | 109.67 | 30.67 | -72.03 | 101.67 | +100 |
| Central | 81.71 | 36.86 | -54.89 | 65.29 | +77.13 |
| Eastern | 52.2 | 11.2 | -78.54 | 54.8 | +100 |
| Greater Accra | 50.17 | 11.83 | -76.42 | 52.67 | +100 |
| North East | 167.5 | 4 | -97.61 | 189 | +100 |
| Northern | 86 | 6 | -93.02 | 129.14 | +100 |
| Oti | 88.75 | 35 | -60.56 | 77.25 | +100 |
| Savannah | 42 | 20.5 | -51.19 | 54.5 | +100 |
| Upper East | 89 | 14.88 | -83.28 | 77.88 | +100 |
| Upper West | 75.78 | 4.11 | -94.58 | 67.33 | +100 |
| Volta | 120.33 | 32.11 | -73.31 | 105.33 | +100 |
| Western | 117.5 | 23.63 | -79.89 | 119.38 | +100 |
| Western North | 62.5 | 15.25 | -75.60 | 66 | +100 |

*Deliveries Conducted*

Table 4 showed that during the strike period, the average number of deliveries conducted across the regions was very low compared to the average number of deliveries conducted prior to the strike and after the strike. In Northern region, for example, an average of 25 deliveries were conducted prior to the strike. This reduced to 2 during the strike period but increased to 40 after the strike was called off. In terms of percentage change in deliveries conducted, Savannah region reported the lowest decrease (10%), whereas Bono East registered the highest decrease (93.61%). With the exception of Greater Accra region, all the other regions recorded more than 100% increase in mean deliveries after the strike was called relative to the strike period.

Table 4: *Percentage change in the mean deliveries conducted during and after strike*

| **Region** | **Before Strike** | **During Strike** | | **After Strike** | |
|---|---|---|---|---|---|
| | Mean Delivery | Mean Delivery | % Δ from Before Strike | Mean Delivery | % Δ from During Strike |
| Ahafo | 18 | 3 | -83.33 | 25 | +100 |
| Ashanti | 12.33 | 1.45 | -88.24 | 10.67 | +100 |
| Bono | 17.83 | 6.17 | -65.40 | 16.33 | +100 |
| Bono East | 11 | 0.67 | -93.91 | 12.67 | +100 |
| Central | 12.43 | 3.43 | -72.40 | 10.29 | +100 |
| Eastern | 14.4 | 3.4 | -76.39 | 12.8 | +100 |
| Greater Accra | 22.17 | 8.83 | -60.17 | 16.83 | 90.60 |
| North East | 19.5 | 4.5 | -76.92 | 20 | +100 |
| Northern | 25.71 | 2 | -92.22 | 40 | +100 |
| Oti | 9.5 | 6.25 | -34.21 | 14 | +100 |
| Savannah | 5 | 4.5 | -10 | 12 | +100 |
| Upper East | 38.5 | 5.38 | -86.03 | 17 | +100 |
| Upper West | 16.44 | 5 | -69.59 | 17 | +100 |
| Volta | 12.33 | 3 | -75.67 | 11.22 | +100 |
| Western | 12.13 | 5.63 | -53.59 | 13 | +100 |
| Western North | 7.75 | 1.25 | -83.87 | 9 | +100 |

*Surgeries Performed*

The data presented in Table 5 revealed that, the average number of surgeries performed decreased across the regions during the strike period, ranging from 67.27% (Savannah region) to 100% (e.g., North East). As can be seen in Table 5, no surgeries were performed in Ahafo and North East regions during the strike period. Whereas there was an increase in surgeries performed in most regions when the strike was called off relative to the strike period (≥ 100% percentage increase in mean), only Savannah region recorded a decrease of 55.56%.

Table 5: *Percentage change in the mean surgeries performed during and after strike*

| **Region** | **Before Strike** | **During Strike** | | **After Strike** | |
|---|---|---|---|---|---|
| | Mean Surgeries | Mean Surgeries | % Δ from Before Strike | Mean Surgeries | % Δ from During Strike |
| Ahafo | 1 | 0 | -100 | 2 | 100 |
| Ashanti | 6.25 | 1.75 | -72 | 5.42 | +100 |
| Bono | 18.33 | 4.17 | -77.25 | 19.17 | +100 |
| Bono East | 17.33 | 2.33 | -86.56 | 16 | +100 |
| Central | 7.14 | 1.29 | -81.93 | 6.57 | +100 |
| Eastern | 18 | 1.4 | -92.22 | 23.2 | +100 |
| Greater Accra | 12.67 | 3.33 | -73.71 | 42.5 | +100 |
| North East | 5 | 0 | -100 | 2.5 | 100 |
| Northern | 8.57 | 0.86 | -89.97 | 12.43 | +100 |
| Oti | 5.25 | 1.5 | -71.43 | 4.75 | +100 |
| Savannah | 27.5 | 9 | -67.27 | 4 | -55.56 |
| Upper East | 7.25 | 0.5 | -93.10 | 12 | +100 |
| Upper West | 6.22 | 1.22 | -80.39 | 6.33 | +100 |
| Volta | 8.33 | 0.89 | -89.32 | 8.56 | +100 |
| Western | 10.75 | 2.75 | -74.42 | 10.25 | +100 |
| Western North | 3.75 | 0.75 | -80 | 5.5 | +100 |

*Reproductive Health Services*

Across the regions, it was observed that the average number of people who accessed reproductive health services dwindled dramatically during the strike period, compared with the periods before and after strike (Table 6). In three of the regions (i.e., Ahafo, Central and Northern), no one accessed reproductive health services during the strike period. The decrease in mean reproductive service use during the strike period, relative to before the strike ranges from 45.74% (Bono region) to 99.80% (Volta region). In contrast, the regions experienced a significant increase (i.e., ≥ 60.74 %) in reproductive service use following the suspension of the strike, except in Savannah region where there was 96% decrease.

Table 6: *Percentage change in the mean reproductive service use (RSU) during and after strike*

| **Region** | **Before Strike** | **During Strike** | | **After Strike** | |
|---|---|---|---|---|---|
| | Mean RSU | Mean RSU | % Δ from Before Strike | Mean RSU | % Δ from During Strike |
| Ahafo | 68 | 0 | -100 | 230 | 100 |
| Ashanti | 88.5 | 8.92 | -89.92 | 119.5 | +100 |
| Bono | 43 | 23.33 | -45.74 | 37.5 | 60.74 |
| Bono East | 63.67 | 5 | -92.15 | 48.67 | +100 |
| Central | 66.5 | 0 | -100 | 55.86 | 100 |
| Eastern | 21.6 | 0.4 | -98.15 | 59.8 | +100 |
| Greater Accra | 78.83 | 13.5 | -82.87 | 76.17 | +100 |
| North East | 107 | 0 | -100 | 161.5 | 100 |
| Northern | 38.33 | 0 | -100 | 60.33 | 100 |
| Oti | 27 | 1 | -96.30 | 37.75 | +100 |
| Savannah | 14 | 25 | 78.57 | 1 | -96 |
| Upper East | 51.75 | 6 | -88.41 | 55.38 | +100 |
| Upper West | 33.56 | 1.11 | -96.69 | 31.89 | +100 |
| Volta | 77.78 | 0.11 | -99.86 | 66.56 | +100 |
| Western | 58.25 | 2.25 | -96.14 | 65 | +100 |
| Western North | 59.5 | 16.25 | -72.69 | 53.25 | +100 |

*Antenatal Clinic Services*

The average number of people who accessed antenatal clinic (ANC) services decreased greatly during the period of strike, compared with the period before and after the strike. Ashanti region recorded the least percentage decrease in mean antenatal use (76.13%), whereas North East and Northern regions registered the highest decrease (100%). Relatedly, across the regions, there was more than 100% increase in antenatal service use following the suspension of the strike action, relative to during the strike.

*Table 7: Percentage change in the mean antenatal clinic service use (ANC) during and after strike*

| **Region** | **Before Strike** | **During Strike** | | **After Strike** | |
|---|---|---|---|---|---|
| | Mean ASU | Mean ASU | % Δ from Before Strike | Mean ASU | % Δ from During Strike |
| Ahafo | 135 | 9 | -93.33 | 175 | +100 |
| Ashanti | 68.42 | 16.33 | -76.13 | 79.67 | +100 |
| Bono | 89 | 13.17 | -85.20 | 115.17 | +100 |
| Bono East | 215 | 67.67 | -68.53 | 234.33 | +100 |
| Central | 58.43 | 6.57 | -88.76 | 70.43 | +100 |
| Eastern | 63.2 | 2.2 | -96.52 | 124.6 | +100 |
| Greater Accra | 87.83 | 19.67 | -77.60 | 98 | +100 |
| North East | 150 | 0 | -100 | 187 | +100 |
| Northern | 164 | 0 | -100 | 291.71 | +100 |
| Oti | 60 | 16.75 | -72.08 | 70.75 | +100 |
| Savannah | 41.5 | 4.5 | -89.16 | 37.5 | +100 |
| Upper East | 27 | 0.5 | -98.15 | 30.25 | +100 |
| Upper West | 29.89 | 0.78 | -97.39 | 39.22 | +100 |
| Volta | 85.33 | 0.75 | -99.12 | 105.67 | +100 |
| Western | 104.5 | 24.88 | -76.19 | 117.5 | +100 |
| Western North | 34.5 | 1 | -97.10 | 48 | +100 |

## Access to Health Services at Polyclinics during Strike

*Outpatient Department Attendance*

The data on OPD attendance in Table 8 revealed that the average number of people accessing OPD services reduced drastically during the strike period, compared with before strike. The percentage decrease in OPD attendance ranges from 23.44% (Central region) to 100% in Bono region. Taking Ashanti region as example, before the strike, 299 people accessed OPD services. However, this dropped to 55 during the strike period, representing 81.61% decrease. The results further showed that, OPD attendance increased above 100% across the regions when the strike was called off.

Table 8: *Percentage change in outpatient department attendance during and after strike*

| **Region** | **Before Strike** | **During Strike** | | **After Strike** | |
|---|---|---|---|---|---|
| | Mean OPD Attendance | Mean OPD Attendance | % Δ from Before Strike | Mean OPD Attendance | % Δ from During Strike |
| Ashanti | 299 | 55 | -81.61 | 409 | +100 |
| Bono | 102 | 0 | -100 | 110 | +100 |
| Central | 128 | 98 | -23.44 | 133 | +100 |
| Greater Accra* | 66 | 7.5 | -88.64 | 38.5 | +100 |
| North East | 142 | 0 | -100 | 144 | +100 |
| Upper West | 65 | 8 | -87.69 | 62 | +100 |
| Western North | 17 | 24 | 41.18 | 14 | +100 |

**= mean/average data*

*Admissions*

As shown in Table 9, the average number of patients on admissions declined during the strike period across the regions. For example, In Bono, Greater Accra and North East regions, there were zero admissions during the strike period. All the regions witnessed an increase in admissions after the strike, ranging from 41.67% to +100%.

Table 9: *Percentage change in mean admissions during and after strike*

| Region | Before Strike | During Strike | | After Strike | |
|---|---|---|---|---|---|
| | Mean Admissions | Mean Admissions | % Δ from Before Strike | Mean Admissions | % Δ from During Strike |
| Ashanti | 29 | 10 | -65.52 | 42 | +100 |
| Bono | 4 | 0 | -100 | 9 | +100 |
| Central | 20 | 12 | -40 | 7 | 41.67 |
| Greater Accra* | 13 | 0 | -100 | 4 | +100 |
| North East | 18 | 0 | -100 | 2 | +100 |

**= mean/average data*

*Deliveries Conducted*

The average number of deliveries conducted reduced during the strike period, compared with the period before and after the strike (Table 10). In Ashanti region, for instance, 2 deliveries were conducted on average during the strike, whereas the average number of deliveries before and after the strike stood at 5 and 15, respectively. In regions such as Bono and North East, there were no deliveries performed during the strike period. However, all the regions recorded more than 100% increase in deliveries conducted after the strike was called off.

Table 10: *Percentage change in deliveries conducted during and after strike*

| Region | Before Strike | During Strike | | After Strike | |
|---|---|---|---|---|---|
| | Mean Delivery | Mean Delivery | % Δ from Before Strike | Mean Delivery | % Δ from During Strike |
| Ashanti | 5 | 2 | -60 | 15 | +100 |
| Bono | 5 | 0 | -100 | 4 | +100 |
| Central | 4 | 3 | -25 | 8 | +100 |
| Greater Accra* | 1.5 | 0.5 | -66.67 | 0 | +100 |
| North East | 8 | 0 | -100 | 5 | +100 |
| Upper West | 3 | 2 | -33.33 | 4 | +100 |

*= mean/average data*

*Reproductive Health Services*

The result in Table 11 showed that, during the strike, no reproductive services were provided at Ashanti, Bono, Greater Accra and Upper West regions. Only Central region provided reproductive health services during the strike period. In Ashanti and Bono regions, more reproductive services were provided after the strike was called off, compared with before the strike was declared.

Table 11: *Percentage change in reproductive service use (RSU) during and after strike*

| **Region** | **Before Strike** | **During Strike** | | **After Strike** | |
|---|---|---|---|---|---|
| | Mean RSU | Mean RSU | % Δ from Before Strike | Mean RSU | % Δ from During Strike |
| Ashanti | 256 | 0 | -100 | 251 | +100 |
| Bono | 8 | 0 | -100 | 12 | +100 |
| Central | 2 | 5 | +100 | 4 | -20 |
| Greater Accra* | 40 | 0 | -100 | 37 | +100 |
| Upper West | 3 | 0 | -100 | 2 | +100 |

*= mean/average data*

## Access to Health Services at Health Centers During Strike

*Outpatient Department Attendance*

As shown in Table 12, the average number of OPD attendance at health centers across the regions decreased during the strike period. Three of the regions (e.g., Greater Accra, Northern and Western regions) recorded no OPD attendance during the strike. For the remaining regions, the percentage decrease in average OPD attendance ranges from 46.86% (Western North region) to 94.49% (Upper West). Similarly, there was over 100% increase in the average OPD when the strike was called off.

Table 12: *Percentage change in the mean OPD attendance during and after strike*

| **Region** | **Before Strike** | **During Strike** | | **After Strike** | |
|---|---|---|---|---|---|
| | Mean OPD Attendance | Mean OPD Attendance | % Δ from Before Strike | Mean OPD Attendance | % Δ from During Strike |
| Ashanti | 44.25 | 11.08 | -74.96 | 48.92 | +100 |
| Bono | 66 | 12 | -81.82 | 132 | +100 |
| Central | 53.62 | 5.92 | -88.96 | 55.23 | +100 |
| Greater Accra | 36 | 0 | -100 | 31 | +100 |
| North East | 105.33 | 33 | -68.67 | 111.33 | +100 |
| Northern | 16 | 0 | -100 | 12 | +100 |
| Savannah | 10.83 | 3 | -72.30 | 12.17 | +100 |
| Upper East | 40.5 | 2.75 | -93.21 | 37.75 | +100 |
| Upper West | 31.75 | 1.75 | -94.49 | 24.25 | +100 |
| Volta | 82 | 14.5 | -82.32 | 94.5 | +100 |
| Western | 18.67 | 0 | -100 | 26 | +100 |
| Western North | 28.6 | 15.2 | -46.85 | 36.2 | +100 |

*Deliveries Conducted*

Table 13 showed that the average deliveries conducted in the health centers reduced significantly during the strike period. The percentage decrease ranges from 14.29% (Western North) to 100% (e.g., Northern region). Following the suspension of the strike, the average

deliveries conducted increased beyond 100% from during the strike period for all but one region (Northern region) where there was no deliveries conducted.

Table 13: *Percentage change in the mean deliveries conducted during and after strike*

| **Region** | **Before Strike** | **During Strike** | | **After Strike** | |
|---|---|---|---|---|---|
| | Mean Deliveries | Mean Deliveries | % Δ from Before Strike | Mean Deliveries | % Δ from During Strike |
| Ashanti | 2.75 | 0.58 | -78.91 | 2.58 | +100 |
| Bono | 0.5 | 0 | -100 | 1 | 100 |
| Central | 1.69 | 0.67 | -60.36 | 2.69 | +100 |
| Greater Accra | 1 | 0.5 | -50 | 4 | +100 |
| North East | 0.83 | 0.17 | -79.52 | 1 | 100 |
| Northern | 1 | 0 | -100 | 0 | 0 |
| Savannah | 1.17 | 1.5 | 28.21 | 1 | +100 |
| Upper East | 1.75 | 0.75 | -57.14 | 1.5 | +75 |
| Upper West | 0.5 | 0.38 | -24 | 0.75 | +100 |
| Volta | 2 | 0 | -100 | 3.5 | +100 |
| Western | 1.33 | 0 | -100 | 0.33 | +100 |
| Western North | 1.4 | 1.2 | -14.29 | 1.6 | +33 |

## Discusion

Nurses and midwives are extremely important in the provision of healthcare services in Ghana, as stated previously. The health system is functional and operational due to the contributions of nurses and midwives. As a result, the strike action embarked on by the GRNMA from 21st septermber 2020 to 23rd September 2020 had significant impacts on access to health care services.

The results of the study showed that access to healthcare services decreased significantly across health facilities during the strike period. The average number of patients on admission before and after strike were more than twice the average number during the strike period. The reduction in healthcare deliveries was evident across the range of healthcare services investigated, including OPD attendance, admissions, deliveries, and surgeries. The findings reported here largely support previous studies from other African countries, including Kenya (Njuguna, 2015), Nigeria (Oleribe et al., 2018) and Egypt (Shukla, 2012) that showed a reduction in healthcare services utilization following strike actions by nurses and other health professionals.

A major strength of the current study is the wider focus. Unlike the narrowed focus of previous studies (Oleribe et al., 2016; Shukla, 2012; Waithaka et al., 2020), this study focused on the impact of strike on a range of healthcare services from the 16 administrative regions of Ghana. Across the regions, there was a significant reduction in reproductive health service utilization. In three of the regions (i.e., Ahafo, Central and Northern), no one accessed reproductive health services during the strike period. The average number of people who utilized antenatal services decreased during the period of strike, compared with the period before and after the strike. In the North East and Northern regions, for example, there was a zero use of antenatal services during the strike. Our study has shown that strike action by nurses and midwives has the propensity to

negatively impact access to a range of healthcare services provided by different units or departments in different administrative or geographical locations.

The decrease in healthcare access could be attributed to the fact that nurses and midwives are the “basic unit” of the healthcare system such that in their absence, the public is not able to initiate help-seeking behavior. Indeed, nurses activate the healthcare system through the provision of basic health services such as temperature, blood pressure and blood glucose that form the foundation for advanced and detailed healthcare services. Besides, nurses provide major support to other health professionals in rendering healthcare services. Surgeries, for instance, cannot be performed by only surgeons without the professional skills of theatre nurses. Surgical cases require admissions before and after the surgical procedure. Nurses generally manage the admission processes. Without adequate number of nurses to care for patients before and after surgery, undesirable surgical outcomes, including complication and wound infection, may manifest quite easily and quickly. Therefore, the absence of nurses tends to create a significant professional gap that other health professionals, by virtue of their training and specialization, are not able to immediately and appropriately fulfill their roles. Because the public is aware of the duties and responsibilities nurses and midwives play in the health system, news about their strike could affect help-seeking decision from healthcare facilities.

The reduction in access to healthcare services have enormous consequences, as demonstrated by previous studies. For example, strike action by healthcare workers has been linked to increased mortalities (Kaguthi et al., 2020). The absence or limited supply of vaccination services occasioned by strike action (Njuguna, 2015) could complicate efforts to improve the health of children and adolescent as well as preventing death from childhood diseases. Although data were not obtained on service users; however, previous studies have shown that social and

economically disadvantaged groups are extremely vulnerable to the negative repercussions of strike as they are unable to pay for services in a private hospital (Irimu et al., 2018). These individuals may resort to the use of herbal preparations and self-medication that often exacerbate their conditions, culminating into increased morbidity and mortality (Irimu et al., 2018; Oleribe et al., 2018; Waithaka et al., 2020).

A major caveat in the literature is the limited data on access to healthcare services following the suspension of strike action by healthcare professionals. To fill this void, the current study compared healthcare services utilization during and after the strike action by nurses and midwives. As reported previously, service used increased drastically following the cessation of the strike action. The increase in access to healthcare services is a testament of the unmet health needs of the public. The decrease in the provision of healthcare services during the strike period could drive the public towards private healthcare system. However, most Ghanaians cannot afford the services of private healthcare facilities owing to the relatively high cost of treatment(Burger et al., 2012). These individuals are left with the option of waiting on the news of resumption of work by nurses and midwives so that they can access healthcare. The drastic increase in the use of healthcare services could be due to several factors, including the fear of another strike. Indeed, the strike was called off following an injunction order secured from a court of competent jurisdiction by the National Labor Commission (NLC) of Ghana, who questioned the legitimacy of the strike. The NLC, which is mandated to address labor agitation and unrest issues in Ghana, does not negotiate with striking labor unions. Like in other jurisdiction, the labor laws favor strike. However, the processes to do so have always been so onerous and time-consuming that unions in Ghana rarely engage in legally recognized strikes (Baah & Reilly, 2009). The public could be worried that the negotiation between the NLC and GRNMA may fail, thereby providing the motivation for another

strike action. Indeed, instances of failed negotiation between the NLC and labor unions are very common development in Ghana. The public, therefore, saw the immediate resumption of duty as the best opportunity to obtain healthcare services before another strike is announced. There is also the possibility that people visited the health facilities to learn for themselves whether the healthcare delivery process has normalized, following its truncation by the strike actions. In the process, they might have accessed healthcare services, adding to the number of service users after the strike period.

**Limitations of the Study**

The study findings should be evaluated considering the following limitations. The study was designed to gather data from the health facilities located across the 16 administrative regions of Ghana. However, as a typical nature of research, we could not obtain data from all the facilities across the regions or districts. Some heads of facilities did not grant approval for the data gathering process. In some instances, we could not identify individuals interested in supporting the research as field officers. The data reported here were based on facilities that were somewhat conveniently selected. The lack of random selection of the health facilities limits the generalization of the findings reported in this study. Given the nature of the research design, particularly the data collection method and data collected, it was impossible to determine whether and to what extent other factors beyond the strike action contributed to the decrease in access to healthcare during strike period and an increase post-strike.

**Conclusions**

The strike action by the nurses and midwives in Ghana significantly reduced access to a range of health services, including reproductive health services, deliveries, and antenatal services.

The distribution of the impacts of the strike action across the regions is largely uniform. Likewise, the impact of the strike action extended to various types of health facilities, hospitals, polyclinics and health centers. The far-reaching impact suggest clearly that strike action by nurses and midwives will derail the efforts and investment made towards achieving the universal healthcare coverage by the Government of Ghana and development partners. It is also imperative to state that the attainment of the goal 3 (health and well-being) of SDG in Ghana will be negatively affected with strike action by nurses and midwives. This understanding calls for proactive measures by the Government of Ghana and relevant organizations to respond adequately and appropriately to the needs and concerns of nurses and midwives as the largest healthcare workforce. Doing so requires some practical steps such as periodic assessment of the wellbeing and conditions of service of nurses and midwives and provide the necessary support system that accommodate existing socioeconomic conditions. Increasing cost of goods and services, for example, should be accompanied by a corresponding increase in salary or financial incentives. This basic economic principle would mitigate against the financial challenges suffered by nurses and midwives, significantly reducing agitation for improved conditions of services amidst economic hardship induced by the raising inflation and cost of petroleum products.

## References

Agyepong, I. A., Kodua, A., Adjei, S., & Adam, T. (2012). When 'solutions of yesterday become problems of today': crisis-ridden decision making in a complex adaptive system (CAS)—the Additional Duty Hours Allowance in Ghana. *Health Policy and Planning*, *27*(suppl_4), iv20-iv31.

Aro, S., & Hosia, P. (1987). Effects of a doctors' strike on primary care utilization in Varkaus, Finland. *Scandinavian Journal of Primary Health Care*, *5*(4), 245-251.

Baah, A. Y., & Reilly, B. (2009). An empirical analysis of strike durations in Ghana from 1980 to 2004. *Labour*, *23*(3), 459-479.

Bhuiyan, M., & Machowski, A. (2012). Impact of 20-day strike in Polokwane hospital (18 August-6 September 2010). *South African Medical Journal*, *102*(9), 755-756.

Burger, N. E., Kopf, D., Spreng, C. P., Yoong, J., & Sood, N. (2012). Healthy firms: constraints to growth among private health sector facilities in Ghana and Kenya. *PloS one*, *7*(2), e27885.

Furnivall, D., Bottle, A., & Aylin, P. (2018). Retrospective analysis of the national impact of industrial action by English junior doctors in 2016. *BMJ Open*, *8*(1), e019319.

Ghana Health Service[GHS]. (2003). *The Status of Referrals in Three Districts in Ghana: Analysis of Referral Pathways for Children under Five. The Basic Support for Institutionalizing Child Survival Project (BASICS II) for the United States Agency for International Development, and Ghana Health Service*.

Ghana Health Service [GHS]. (2018). *articles The Health Sector in Ghana. Facts and Figures 2018om*. https://open.africa/dataset/4176f749-cfa8-4e32-9418-86cef78f9db6/resource/0bcf9b54-3e35-4543-95cd-fd4de953edff/download/factsfigures_2018.pdf

Gyamfi, G. D. (2011). Assessing the effects of industrial unrest on Ghana health service: A case study of nurses at Korle-Bu teaching hospital. *International Journal of Nursing and Midwifery*, *3*(1), 1-5.

Irimu, G., Ogero, M., Mbevi, G., Kariuki, C., Gathara, D., Akech, S., Barasa, E., Tsofa, B., & English, M. (2018). Tackling health professionals' strikes: an essential part of health system strengthening in Kenya. *BMJ Global Health*, *3*(6).

Isangula, K. (2012). Activism during doctor's strike in Tanzania, success and setbacks. SSRN Electronic Journal *Sikika Journal*. Available a http://dx.doi.org/10.2139/ssrn.2129021

Kaguthi, G. K., Nduba, V., & Adam, M. B. (2020). The impact of the nurses', doctors' and clinical officer strikes on mortality in four health facilities in Kenya. *BMC Health Services Research*, *20*(1), 1-10.

Manthous, C. A. (2014). Labor unions in medicine: The intersection of patient advocacy and self-advocacy. *Medical care*, *52*(5), 387-392.

Njuguna, J. (2015). Impact of health workers' strike in August 2014 on health services in Mombasa County Referral Hospital, Kenya. *Journal of Health Care for the Poor And Underserved*, *26*(4), 1200-1206.

Oleribe, O. O., Ezieme, I. P., Oladipo, O., Akinola, E. P., Udofia, D., & Taylor-Robinson, S. D. (2016). Industrial action by healthcare workers in Nigeria in 2013–2015: an inquiry into causes, consequences and control—a cross-sectional descriptive study. *Human resources for Health*, *14*(1), 1-10.

Oleribe, O. O., Udofia, D., Oladipo, O., Ishola, T. A., & Taylor-Robinson, S. D. (2018). Healthcare workers' industrial action in Nigeria: a cross-sectional survey of Nigerian physicians. *Human Resources for Health*, *16*(1), 1-10.

Ruiz, M., Bottle, A., & Aylin, P. (2013). A retrospective study of the impact of the doctors' strike in England on 21 June 2012. *Journal of the Royal Society of Medicine*, *106*(9), 362-369.

Scanlon, M. L., Maldonado, L. Y., Ikemeri, J. E., Jumah, A., Anusu, G., Chelagat, S., Keter, J. C., Songok, J., Ruhl, L. J., & Christoffersen-Deb, A. (2021). 'It was hell in the community': a qualitative study of maternal and child health care during health care worker strikes in Kenya. *International Journal for Equity in Health*, *20*(1), 1-12.

Sheahan, K. H., Kennedy, A. G., Tompkins, B. J., Repp, A. B., & Gilbert, M. P. (2021). The Impact of a Nursing Strike on Glycemic Control in Hospitalized Patients with Diabetes. *Cureus*, *13*(6).

Shukla, S. (2012). Violence against doctors in Egypt leads to strike action. *The Lancet*, *380*(9852), 1460.

Van Rensburg, A. J., & van Rensburg, D. J. (2013). Nurses, industrial action and ethics: considerations from the 2010 South African public-sector strike. *Nursing Ethics*, *20*(7), 819-837.

Waithaka, D., Kagwanja, N., Nzinga, J., Tsofa, B., Leli, H., Mataza, C., Nyaguara, A., Bejon, P., Gilson, L., & Barasa, E. (2020). Prolonged health worker strikes in Kenya-perspectives and experiences of frontline health managers and local communities in Kilifi County. *International Journal for Equity in Health*, *19*(1), 23.

Supplementary Files

File 1: Raw data used for the analysis.

**Response to Reviewer**

**PONE-D-21-35187**

We are grateful for the feedback on our manuscript. We have responded to the issues raised in the revised manuscript.

We have re-worded the title from *"Macro-level Impact of Strike Action by Ghana Registered Nurses and Midwives"* to *"A Multi-Level Impact Analysis of Strike Action by Ghana Registered Nurses and Midwives on Access to and Utilization of Healthcare Services"*

We have highlighted our responses in yellow ink for quick reference.

Reviewer #1: Overall
-Interesting study that shows the lack of access to care for the public when healthcare workers go on strike. See below a few suggestions, and questions for clarification to improve how the story is presented.

Authors response: Thank you very much for the feedback.

Title
**Reviewer comment** -Suggest, rewording the title, as it does not only focus on the macro level of the health system (which I understand to be the national level). The study focuses on hospitals and health centres across different regions suggesting a multi-level approach.

Authors response: Thank you very much. We have revised the title as suggested.

Abstract
Reviewer comment-Suggest similar wording in the abstract regarding how many facilities were accessed for data collection as in the methods section (see comment on article).

Authors response: Thank you. This has been revised.

Introduction and Background
**Reviewer comment**-The introduction needs improvement to have better flow. Currently the integration mixes information about HCW strikes from various strikes across different African countries, thus it is not quite clear what gap this specific paper is filling in the already existing literature, or what exactly it will be adding on. I suggest re-ordering of the information presented starting with what is known about HCW strikes worldwide and their effects, what is known in Africa, and then in Ghana, then stating which areas the literature (especially from Africa and Ghana) has paid less attention to, and then stating as has been well-stated in the paper what gaps the paper will be filling.

Authors response: We are grateful for this observation. We have revised the manuscript and incorporated the suggestions you offered. Thank you.

**Reviewer comment**-Would also be useful to tell the reader a bit more about the Ghana health system context. This is included in the project setting, but suggest moving to the introduction

Authors response: We have done so accordingly.

**Reviewer comment**-Suggest carefully re-reading of the introduction to ensure adequate referencing of studies e.g. the sentence on the longest ever strike action needs referencing. Other statements about studies done elsewhere reporting on low immunization rates during HCW strikes need citation too (pgs 4-6).

Authors response: We have effected this recommendation as well. Thank you very much.

**Reviewer comment**-Also re-read to check typos and sentence structure to improve clarity. See comments on the paper

Authors response: Thank you very much for the comments. We have improved on the manuscript in this regard.

Methods

**Reviewer comment** -Project setting section states data was collected from 155 government-owned facilities while results report on data from 188 facilities. What of the other remaining facilities, who owned them?

**Authors response:** Thank you for the observation. We collected data from health facilities that were categorized as Government and CHAG. Those falling under government were 155, whereas the remining 26 were CHAG facilities. We have explained this further in the revised manuscript.

Reviewer comment -Not clear what/which hospitals or health centres were included for every region. Was it every hospital and/or health centre in the region. If not, what was the selection criteria for the health facilities selected for data collection?

Authors response: Thank you very much. We designed the study to gather data from all health facilities located in each region or district. We did not aim to exclude any health facility as we wanted to understand the impact of strike across the levels of healthcare

delivery and across regions or districts. However, as a typical nature of research, we could not obtain data from all the facilities across the regions or districts. Some heads of facilities did not grant approval for the data gathering process. In some instances, we could not identify individuals interested in supporting the research as field officers. The data obtained were what we reported. This shortfall is acknowledged in the limitation section of the study.

**Reviewer comment** -Suggest moving the information on the Ghana health system (first three sentences could be moved to the introduction part)

Authors response: Thank you very much. We have included a brief summary on the organization of healthcare system in Ghana to enable readers contextualized the project. We are grateful for the comments.

**Reviewer comment** -Some of the information on the section on training field officers is repetitive. Suggest summarising in one or two sentences and re-wording for simplicity, for example by mentioning what the field officers were trained on, and on which platform training occurred. Overall suggest reduction of information about the field officers (e.g. information on how much they were paid etc not necessary).

Authors response: We are grateful for the comment. The manuscript has been revised accordingly by simplifying the information on the field officers.

**Reviewer comment** -Regarding your variables, aren't reproductive health services inclusive of ANC, and delivery services? Did reproductive health services only refer to family planning services? If this is the case, then I suggest to use the term family planning services instead for clarity.

Authors response: Thank you very much for the observation and comments. Yes, in some instances such as during pregnancy, reproductive health issues are integral component of ANC services. On the other hand, reproductive health services are provided to persons who do not require ANC services. For example, non-pregnant women do access reproductive health services including family planning and pre-pregnancy support services. It is against this basis that we separated ANC services from reproductive health services. We made this clear in the revised manuscript.

**Reviewer comment** -Curious why immunisation services were not included as among the variables reported in the study? In most Low and Middle Income Countries (LMICs) these

are provided by nurses and have been shown to be affected when healthcare workers' strikes occur.

Authors response: This is a very important observation. In fact, it was an oversight. In future endeavors, we would certainly include immunization or vaccination services.

Results

**Reviewer comment** -Suggest a brief introduction that summarises what results will be presented. This could also include a sentence reminding the reader that the data on hospitals will be presented separately.

Authors response: Thank you very much. We have incorporated this suggestion into the revised manuscript.

Discussion

**Reviewer comment** -Discussion refers to decline in child health services, yet these are not reported on in the results section. Please clarify which child health services were affected…are these immunisation services? Or consultation for children with illnesses in the OPD? If the latter, important to make this distinction in the results section

Authors response: We noted this comment very well. We agreed that we over-stretched the findings. The manuscript has been revised accordingly. We have removed any indication of child health services or immunization services from the revised manuscript.

Reviewer comment -The discussion also refers to the decline in surgical services during the strike and links this to mortalities, however no mortality data has been reported. The data does not show us a link between the fewer surgeries conducted and mortality, either from this study or other published literature. It's necessary to justify this claim, especially because a few studies have shown the absence of a significant increase in mortality during healthcare worker strike periods (see Ong'ayo, Gerald, et al. "Effect of strikes by health workers on mortality between 2010 and 2016 in Kilifi, Kenya: a population-based cohort analysis." The Lancet Global Health 7.7 (2019): e961-e967; Cunningham, Solveig Argeseanu, et al. "Doctors' strikes and mortality: a review." Social science & medicine 67.11 (2008): 1784-1788.)

Authors response: Thank you for the comment. We have delinked any mortality discussion from surgical services. We did not have data on mortality to inform our discussions.

**Reviewer comment** -One of the study limitations was the lack of random selection of facilities for inclusion in this study. However, it has not been clarified, here or in the methods section, how the facilities were selected

Authors response: Thank you for the feedback. This has been included in the revised manuscript, under the limitations.

**Reviewer comment** -The discussion hardly interprets the study findings in the context of other existing literature on healthcare worker strikes, either in Ghana or other parts of the world. I suggest that some of the papers referred to in the introduction could be revisited in the discussion to compare their findings and this study's findings including what new angle, the study adds to our understanding of healthcare worker strikes and their effects. If there are differences between existing study and other literature, then useful to suggest reasons why this might be the case.

Authors response: The discussion session has been revised accordingly.
