# Supplementary material for: The impact of strike action by Ghana registered nurses and midwives on the access to and utilization of healthcare services
Source: PLoS One. 2022 Oct 14;17(10):e0275661. doi: 10.1371/journal.pone.0275661 (PMC9565728; doi:10.1371/journal.pone.0275661)
Supplement: Attachment — Submitted filename: Response to Reviewers.docx [file pone.0275661.s003.docx]

**Response to Reviewer**

**PONE-D-21-35187**

We are grateful for the feedback on our manuscript. We have responded to the issues raised in the revised manuscript.

We have re-worded the title from *“Macro-level Impact of Strike Action by Ghana Registered Nurses and Midwives*” to “*A Multi-Level Impact Analysis of Strike Action by Ghana Registered Nurses and Midwives on Access to and Utilization of Healthcare Services*”

We have highlighted our responses in yellow ink for quick reference.

Reviewer #1: Overall
-Interesting study that shows the lack of access to care for the public when healthcare workers go on strike. See below a few suggestions, and questions for clarification to improve how the story is presented.

Authors response: Thank you very much for the feedback.

Title
**Reviewer comment** -Suggest, rewording the title, as it does not only focus on the macro level of the health system (which I understand to be the national level). The study focuses on hospitals and health centres across different regions suggesting a multi-level approach.

Authors response: Thank you very much. We have revised the title as suggested.

Abstract
Reviewer comment-Suggest similar wording in the abstract regarding how many facilities were accessed for data collection as in the methods section (see comment on article).

Authors response: Thank you. This has been revised.

Introduction and Background
**Reviewer comment**-The introduction needs improvement to have better flow. Currently the integration mixes information about HCW strikes from various strikes across different African countries, thus it is not quite clear what gap this specific paper is filling in the already existing literature, or what exactly it will be adding on. I suggest re-ordering of the information presented starting with what is known about HCW strikes worldwide and their effects, what is known in Africa, and then in Ghana, then stating which areas the literature (especially from Africa and Ghana) has paid less attention to, and then stating as has been well-stated in the paper what gaps the paper will be filling.

Authors response: We are grateful for this observation. We have revised the manuscript and incorporated the suggestions you offered. Thank you.

**Reviewer comment**-Would also be useful to tell the reader a bit more about the Ghana health system context. This is included in the project setting, but suggest moving to the introduction

Authors response: We have done so accordingly.

**Reviewer comment**-Suggest carefully re-reading of the introduction to ensure adequate referencing of studies e.g. the sentence on the longest ever strike action needs referencing. Other statements about studies done elsewhere reporting on low immunization rates during HCW strikes need citation too (pgs 4-6).

Authors response: We have effected this recommendation as well. Thank you very much.

**Reviewer comment**-Also re-read to check typos and sentence structure to improve clarity. See comments on the paper

Authors response: Thank you very much for the comments. We have improved on the manuscript in this regard.

Methods
**Reviewer comment** -Project setting section states data was collected from 155 government-owned facilities while results report on data from 188 facilities. What of the other remaining facilities, who owned them?

**Authors response:** Thank you for the observation. We collected data from health facilities that were categorized as Government and CHAG. Those falling under government were 155, whereas the remining 26 were CHAG facilities. We have explained this further in the revised manuscript.

Reviewer comment -Not clear what/which hospitals or health centres were included for every region. Was it every hospital and/or health centre in the region. If not, what was the selection criteria for the health facilities selected for data collection?

Authors response: Thank you very much. We designed the study to gather data from all health facilities located in each region or district. We did not aim to exclude any health facility as we wanted to understand the impact of strike across the levels of healthcare delivery and across regions or districts. However, as a typical nature of research, we could not obtain data from all the facilities across the regions or districts. Some heads of facilities did not grant approval for the data gathering process. In some instances, we could not identify individuals interested in supporting the research as field officers. The data obtained were what we reported. This shortfall is acknowledged in the limitation section of the study.

**Reviewer comment** -Suggest moving the information on the Ghana health system (first three sentences could be moved to the introduction part)

Authors response: Thank you very much. We have included a brief summary on the organization of healthcare system in Ghana to enable readers contextualized the project. We are grateful for the comments.

**Reviewer comment** -Some of the information on the section on training field officers is repetitive. Suggest summarising in one or two sentences and re-wording for simplicity, for example by mentioning what the field officers were trained on, and on which platform training occurred. Overall suggest reduction of information about the field officers (e.g. information on how much they were paid etc not necessary).

Authors response: We are grateful for the comment. The manuscript has been revised accordingly by simplifying the information on the field officers.

**Reviewer comment** -Regarding your variables, aren’t reproductive health services inclusive of ANC, and delivery services? Did reproductive health services only refer to family planning services? If this is the case, then I suggest to use the term family planning services instead for clarity.

Authors response: Thank you very much for the observation and comments. Yes, in some instances such as during pregnancy, reproductive health issues are integral component of ANC services. On the other hand, reproductive health services are provided to persons who do not require ANC services. For example, non-pregnant women do access reproductive health services including family planning and pre-pregnancy support services. It is against this basis that we separated ANC services from reproductive health services. We made this clear in the revised manuscript.

**Reviewer comment** -Curious why immunisation services were not included as among the variables reported in the study? In most Low and Middle Income Countries (LMICs) these are provided by nurses and have been shown to be affected when healthcare workers’ strikes occur.

Authors response: This is a very important observation. In fact, it was an oversight. In future endeavors, we would certainly include immunization or vaccination services.

Results
**Reviewer comment** -Suggest a brief introduction that summarises what results will be presented. This could also include a sentence reminding the reader that the data on hospitals will be presented separately.

Authors response: Thank you very much. We have incorporated this suggestion into the revised manuscript.

Discussion
**Reviewer comment** -Discussion refers to decline in child health services, yet these are not reported on in the results section. Please clarify which child health services were affected…are these immunisation services? Or consultation for children with illnesses in the OPD? If the latter, important to make this distinction in the results section
Authors response: We noted this comment very well. We agreed that we over-stretched the findings. The manuscript has been revised accordingly. We have removed any indication of child health services or immunization services from the revised manuscript.

Reviewer comment -The discussion also refers to the decline in surgical services during the strike and links this to mortalities, however no mortality data has been reported. The data does not show us a link between the fewer surgeries conducted and mortality, either from this study or other published literature. It’s necessary to justify this claim, especially because a few studies have shown the absence of a significant increase in mortality during healthcare worker strike periods (see Ong'ayo, Gerald, et al. "Effect of strikes by health workers on mortality between 2010 and 2016 in Kilifi, Kenya: a population-based cohort analysis." The Lancet Global Health 7.7 (2019): e961-e967; Cunningham, Solveig Argeseanu, et al. "Doctors' strikes and mortality: a review." Social science & medicine 67.11 (2008): 1784-1788.)

Authors response: Thank you for the comment. We have delinked any mortality discussion from surgical services. We did not have data on mortality to inform our discussions.

**Reviewer comment** -One of the study limitations was the lack of random selection of facilities for inclusion in this study. However, it has not been clarified, here or in the methods section, how the facilities were selected

Authors response: Thank you for the feedback. This has been included in the revised manuscript, under the limitations.

**Reviewer comment** -The discussion hardly interprets the study findings in the context of other existing literature on healthcare worker strikes, either in Ghana or other parts of the world. I suggest that some of the papers referred to in the introduction could be revisited in the discussion to compare their findings and this study’s findings including what new angle, the study adds to our understanding of healthcare worker strikes and their effects. If there are differences between existing study and other literature, then useful to suggest reasons why this might be the case.

Authors response: The discussion session has been revised accordingly.
