# Supplementary material for: The impact of strike action by Ghana registered nurses and midwives on the access to and utilization of healthcare services
Source: PLoS One. 2022 Oct 14;17(10):e0275661. doi: 10.1371/journal.pone.0275661 (PMC9565728; doi:10.1371/journal.pone.0275661)
Supplement: Attachment — Submitted filename: Response to Reviewers Comments.docx [file pone.0275661.s005.docx]

**Comment 1:** We noticed that your manuscript did not have ethical approval possibly because it was a retrospective anonymous data analysis. However, we suggest that you obtain a communication from your local ethics review board that the research do not require an ethical approval and that the dissemination of the research may not violate any ethical principles. You may then adjust your ethics statement to include such communication from ethics committee.

Authors’ response: Thank you very much for the recommendation. We have received a response from the local ethics and have included same in the ethics section of the manuscript.

**Comment 2:** Title: "Multi-level ...." is usually reserved for a specific statistical analysis which was not conducted in this study. It is suggested that the title should be revised so as not to confuse the audience.

Authors response: The title has been modified to “The Impact of Strike Action by Ghana Registered Nurses and Midwives on the Access to and Utilization of Healthcare Services” (Page 1, line 1-2, page 2, lines 27)

**Comment 3**: First objective: Please change "utilization" to "utilizing"

Authors response: The change has been applied. (Page 6, line 140).

**Comment 4**: For ease of review, Please provide a continuous number line while revising your manuscript.

Authors response: This has been provided.

**Comment 5:** Results: Please delete "In this section, we presented the results of the data analyses, commencing with the distribution of healthcare facilities. This was followed by the presentation of findings relating to the utilization of healthcare services. This was structured based on the type of health facilities, starting first with hospitals, followed by polyclinics, and lastly health centers. We examined the extent of and change in use of the various health services described under study variables for the various health facility types. This was done separately for each region"

Authors response: This section has been deleted.

**Comment 6**: References: The references are not in line with the referencing style of PLOS ONE. (See: [Submission Guidelines | PLOS ONE](https://journals.plos.org/plosone/s/submission-guidelines#loc-manuscript-organization))

Authors response: The reference has been formatted in line with the style of PLOS ONE.
